# Family play, reading, and other stimulation and early childhood development in five low‐and‐middle‐income countries

**DOI:** 10.1111/desc.13404

**Published:** 2023-04-28

**Authors:** Jorge Cuartas, Dana McCoy, Juliana Sánchez, Jere Behrman, Claudia Cappa, Georgina Donati, Jody Heymann, Chunling Lu, Abbie Raikes, Nirmala Rao, Linda Richter, Alan Stein, Hirokazu Yoshikawa

**Affiliations:** ^1^ Harvard Graduate School of Education Cambridge Massachusetts USA; ^2^ Centro de Estudio sobre Seguridad y Drogas (CESED) Universidad de los Andes Bogota Colombia; ^3^ Facultad de Economía Universidad de los Andes Bogota Colombia; ^4^ Department of Economics University of Pennsylvania Philadelphia Pennsylvania USA; ^5^ Data and Analytics Section UNICEF New York New York USA; ^6^ Department of Psychiatry University of Oxford Oxford Oxfordshire UK; ^7^ WORLD Policy Analysis Center University of California Los Angeles California USA; ^8^ Brigham & Women's Hospital Harvard Medical School Boston Massachusetts USA; ^9^ College of Public Health University of Nebraska Omaha Nebraska USA; ^10^ Faculty of Education University of Hong Kong Hong Kong China; ^11^ DSI‐NRF Centre of Excellence in Human Development University of the Witwatersrand Johannesburg South Africa; ^12^ Department of Psychiatry University of Oxford Oxford Oxfordshire UK; ^13^ MRC/Wits Rural Public Health and Health Transitions Research Unit (Agincourt) School of Public Health Faculty of Health Sciences University of the Witwatersrand Johannesburg Gauteng South Africa; ^14^ African Health Research Institute Durban South Africa; ^15^ NYU Global TIES for Children Center New York New York USA

**Keywords:** early childhood development, fixed‐effects, home environment, low‐and‐middle‐income countries, stimulation

## Abstract

This paper used longitudinal data from five studies conducted in Bangladesh, Bhutan, Cambodia, Ethiopia, and Rwanda to examine the links between family stimulation and early childhood development outcomes (*N* = 4904; *M*_age_ = 51.5; 49% girls). Results from random‐effects and more conservative child‐fixed effects models indicate that across these studies, family stimulation, measured by caregivers’ engagement in nine activities (e.g., reading, playing, singing), predicted increments in children's early numeracy, literacy, social‐emotional, motor, and executive function skills (standardized associations ranged from 0.05 to 0.11 *SD*). Study‐specific models showed variability in the estimates, with null associations in two out of the five studies. These findings indicate the need for additional research on culturally specific ways in which caregivers may support early development and highlight the importance of promoting family stimulation to catalyze positive developmental trajectories in global contexts.

**Research Highlights:**

- Research on the links between family stimulation and early childhood development in low‐and‐middle‐income countries (LMICs) is limited.
- We used longitudinal data from studies conducted in five LMICs to examine the links between family stimulation and early childhood development outcomes.
- Results suggest that family stimulation predicted increments in children's numeracy, literacy, social‐emotional, motor, and executive function skills.
- We found variability in the observed estimates, with null associations in two out of the five studies, suggesting the need for additional research in LMICs.

## 1 INTRODUCTION

The United Nations’ Sustainable Development Goals include early childhood development as a global priority, specifying that “by 2030… all girls and boys [should] have access to quality early childhood development, care and pre‐primary education so that they are ready for primary education” (United Nations Development Programme, 2015, Target 4.2). Yet, millions of children living in low‐and‐middle‐income countries (LMICs) are estimated to be “developmentally off‐track” and at risk of not realizing their human potential in adulthood (Black et al., 2017; Lu et al., 2020; McCoy et al., 2016). In addition to poverty and stunted growth, insufficient exposure to nurturing caregiving and early stimulation by parents and other caregivers are thought to be major contributors to these developmental challenges (Britto et al., 2017; McCoy et al., 2018; McCoy et al., 2022). The loss in developmental potential in LMICs has profound implications for individuals and societies (Black et al., 2017; Shonkoff & Garner, 2011). It is also a violation of children's rights (UN General Assembly, 1989) and a setback to global policy goals (Black et al., 2017; Shonkoff & Garner, 2011). As a consequence, there is growing interest in promoting early stimulation in families—that is, opportunities for children to engage in reciprocal, playful, and educational interactions with caregivers—as a promising means to promote children's healthy development globally (Britto et al., 2017; Richter et al., 2017).

While existing research shows positive links between early stimulation and early childhood development (ECD; e.g., Cabrera et al., 2020; Jeong et al., 2017), further evidence on the developmental consequences of stimulation in LMICs is needed for advancing developmental theory and supporting advocacy and policy efforts. Most evidence on family stimulation—and indeed, developmental processes in general—comes from high‐income countries (HICs; Draper et al., 2022; Nielsen et al., 2017). Lack of evidence from LMICs is an impediment to generalizable theory, global policy, and appropriate practice (Draper et al., 2022; Nielsen et al., 2017; Rowley & Camacho, 2015). Furthermore, existing studies of stimulation in LMICs (e.g., Jeong et al., 2017; Wolf & McCoy, 2019) have relied primarily on cross‐sectional, correlational designs that make it challenging to understand whether it is stimulation or other characteristics that covary with stimulation and ECD, such as socioeconomic or genetic factors, that leads to better ECD outcomes. Moreover, most comparative research from LMICs employs measures of ECD that are reported by parents and limited in scope, such as the 10‐item Early Childhood Development Index (ECDI; Loizillon et al., 2017). These shortcomings obscure variation in specific developmental domains (e.g., cognitive, motor, social‐emotional) and raise concerns about shared‐method variance.

In the present study, we contribute to the existing body of research on child development in LMICs by assessing the links between caregiver‐reported early stimulation by family members (i.e., adults’ engagement in basic activities such as reading and playing with children in the last week) and preschool‐age children's ECD outcomes using comparable longitudinal data from Bangladesh, Bhutan, Cambodia, Ethiopia, and Rwanda. We attempt to overcome the limitations of prior research by leveraging longitudinal data with more conservative estimation methods that mitigate issues of selection bias. We also employ information from direct assessments of children's skills to assess specific developmental outcomes that might benefit from early stimulation. In addition to these methodological advantages, this study also contributes to developmental theory and ongoing discussions on universality versus specificity of different aspects of parenting (e.g., Bornstein & Putnick, 2012; LeVine et al., 2008; Mesman et al., 2018; Morelli et al., 2017; Rogoff et al., 1993), focusing on a particular component—early stimulation—that is gaining momentum in the global policy agenda.

### 1.1 Conceptual links between stimulation and early childhood development

We employ a bioecological systems perspective as the overarching conceptual framework for this study (Bronfenbrenner & Morris, 2007; Sameroff, 2010). This perspective posits that human development unfolds through dynamic, progressively more complex, reciprocal interactions in the micro‐, meso‐, exo‐, chrono‐, and macrosystems, with proximal processes as the main drivers of children's skill development (Bronfenbrenner & Morris, 2007). Proximal processes encompass interpersonal interactions between children and others that occur repeatedly in the immediate environment. A critical feature of a proximal process is children's active, reciprocal engagements in interaction with adults and peers, as bidirectionality is hypothesized to catalyze skill development (Bronfenbrenner & Morris, 2007; Sameroff, 2010).

Research has shown that proximal processes, in particular caregiver‐child interactions, have direct influences on children's outcomes early in life (Britto et al., 2017). The first years of life are characterized by heightened sensitivity to environmental stimuli and neural and skill development are highly plastic in response to the extent and quality of children's experiences (Berens & Nelson, 2019; Black et al., 2017). For the purpose of this study, we focus on preschool‐aged children and conceptualize their development as a multidimensional set of numeracy, literacy, social‐emotional, motor, and executive function skills that help them adapt to and succeed in the physical and relational world (Pisani et al., 2018). In addition, although we acknowledge that other key sources of interactions include older children and siblings, we focus in this study on primary caregivers.

Recognizing that there are multiple forms of child‐caregiver interactions relevant to the early childhood period (Bornstein, 2015), we concentrate on the provision of early stimulation in families, also described as engagement in learning opportunities (McCoy et al., 2018) or playful learning (Zosh et al., 2017). For the purposes of this paper, we define early stimulation as the various cognitively and social‐emotionally enriching activities that parents and other caregivers engage in with children in or around the home. High‐quality stimulation includes caregivers’ provision of playful environments, modeling, scaffolding, and responsiveness, and provides children with opportunities to engage actively and symbolically in ways that they might not regularly on their own (Bandura, 1986; Bronfenbrenner & Morris, 2007; Vygotsky, 1981). We follow prior research in LMICs (e.g., Jeong et al., 2017; Kariger et al., 2013; Wolf & McCoy, 2019) and operationalize stimulation as child‐caregiver engagement in enriching activities like shared‐book reading, telling stories, singing songs, going outside the home, playing games, naming objects and drawing things, and learning new things (e.g., words, letters, or numbers). These activities are not exclusively learning opportunities for children, but are key interactions—that is, proximal processes—that can be defined as playful—that is, joyful, meaningful, actively engaging, iterative, and socially interactive (Zosh et al., 2017)—and cognitively and social‐emotionally stimulating (Bornstein & Putnick, 2012; McCoy et al., 2018).

Caregivers’ engagement in stimulation activities can promote children's development in multiple domains, including early literacy, numeracy, and motor skill development. For example, activities like reading books, telling stories, singing songs, and playing counting or simple games enable children's early numeracy skills such as category recognition and counting and expose them to novel words, longer utterances, and novel syntax and reasoning (Cabrera et al., 2020; Malin et al., 2014; Rodriguez & Tamis‐LeMonda, 2011; Tamis‐LeMonda et al., 2004; Xiong et al., 2020). Caregivers also employ speech directed to children when reading books, telling stories, playing, or teaching something new, which is an immediate means to convey new information and is a key predictor of language development (Romeo et al., 2018; Rowe, 2008). Other activities like playing, drawing, and going outside the home also expose children to movement behaviors, new people, scenarios, ideas, sounds, and events, where they explore and can be helped to make sense of natural and social phenomena. Together these interactions help to develop abstract thinking, engagement in physical activities, and problem‐solving with support and modeling from caregivers in ways that are beneficial for both cognitive and motor development (Bandura, 1986; Ginsburg et al., 2007; Vygotsky, 1981; Zosh et al., 2017).

In addition to promoting literacy, numeracy, and motor development, stimulation includes interpersonal interactions and opportunities to develop children's social‐emotional and executive function skills. For instance, shared‐book reading, singing songs, playing, and other stimulation activities are interactional, allowing children opportunities to discern and understand others’ emotions, behaviors, and expectations, and to learn to pay attention, engage in problem solving, delay gratification, and follow rules, all of which enhance executive function (Shaheen, 2014; Zosh et al., 2017). For example, in pretend play, children employ their imagination to take on roles in which they set and follow rules and learn how to engage with others, strengthening inhibitory control, as well as social and emotional regulation (Ginsburg et al., 2007; Vygotsky, 1981). Similarly, storytelling, singing songs, and playing are relevant traditions in all cultures to communicate concepts, establish social norms and expectations, and introduce children to valued social dynamics (Bruner, 1986; Roopnarine et al., 1994).

### 1.2 Family stimulation and early childhood development in LMICs

Even though 90% of young children live in LMICs and contexts in LMICs are quite heterogeneous, children living in these countries are severely underrepresented in developmental research (Draper et al., 2022; Nielsen et al., 2017; World Bank, 2020). The nascent literature on family stimulation in LMICs has shown initial evidence for both similarities and differences relative to the HICs‐based literature. Quantitative cross‐cultural studies comprising nationally representative samples of about 160,000 preschool‐age children living in 63 LMICs, revealed that more than 70% experienced “high levels” of family stimulation, defined as engaging in at least four of six measured stimulation activities (e.g., reading, counting, singing) with any adult caregiver (McCoy et al., 2018). Caregivers’ engagement in stimulation is lower, on average, in LMICs relative to HICs, particularly in Sub‐Saharan Africa and parts of Asia, and, within countries, in households with lower wealth relative to wealthier households (Lu et al., 2020; McCoy et al., 2018; Sun et al., 2016). Research also has shown different patterns of who provides stimulation, with LMIC‐based research showing evidence for the active engagement of multiple caregivers in early stimulation activities with children (Cheung et al., 2021; Cuartas, Jeong, et al., 2020), which stands in contrast with much of traditional theory and research based on HICs that has largely focused on mothers (Ainsworth, 1979; Keller et al., 2018; Mesman et al., 2018).

Qualitative and mixed methods research also show both similarities and variation in family stimulation across settings. Reviews of ethnographic, qualitative, and quantitative studies suggest differences in child‐caregiver stimulation activities and the specific manifestations of the same type of activity (Cheung et al., 2021; Nag et al., 2019). For example, there are significant differences in parents’ engagement in child‐led play across Asian countries, likely due to variation in socialization goals, type of activities that are valued, and parents’ attitudes toward free play versus more academically‐focused training and direct instruction (Aminipour et al., 2018; Cheung et al., 2021; Pisani et al., 2016; Shiakou & Belsky, 2013). Collectively, these findings suggest variability in social norms, beliefs, parenting expectations, and family resources (among others) within and between cultures and settings, which demonstrates the importance of studying stimulation in LMICs.

Despite variation in family stimulation within and between countries, existing evidence suggests that stimulation is consistently related to positive ECD outcomes in LMICs. Studies using large‐scale data from Multiple Indicator Cluster Surveys (MICS) conducted in over 38 countries, found that mothers’ and fathers’ reported engagement in six different stimulation activities was positively associated with children's development as measured by the ECDI, a parent‐reported 10‐item measure of early development (Jeong et al., 2017; Jeong et al., 2016). Another investigation of 26 LMICs using MICS found positive associations between adult caregivers’ engagement in stimulation activities and three indicators of early literacy‐numeracy skills (e.g., “Can [name] identify or name at least ten letters of the alphabet?”) and two indicators of social‐emotional skills (e.g., “Does [name] follow simple directions on how to do something correctly?”) from the ECDI (Frongillo et al., 2017). Similar research has used country‐specific MICS or Demographic and Health Surveys (DHS) data, finding consistent positive associations between stimulation and the ECDI in Bangladesh (Alam et al., 2021), Honduras (Urke et al., 2018), Nepal (Sk et al., 2020), and Vietnam (Duc, 2016).

Complementing these large‐scale analyses using simple measures of childhood development, other studies have employed country‐specific data with more comprehensive measures that acknowledge the multidimensionality of ECD and reach similar conclusions. For example, a study in Colombia found positive associations between maternal stimulation and infants’ cognitive, receptive language, and gross motor development as measured by the Bayley Scales of Infant Development (Cuartas, Rey‐Guerra, et al., 2020). Another Colombian study using a national sample of preschoolers (i.e., Rey‐Guerra et al., 2022) identified positive relations between mothers’, fathers’, and other adult caregivers’ stimulation and children's directly assessed emergent numeracy, literacy, social‐emotional, motor, and executive function skills using the International Development and Early Learning Assessment (IDELA; Pisani et al., 2018). Moreover, one study using the China Family Panel Studies found that cognitive stimulation in the home predicted gains in children's cognitive development (Xiong et al., 2020). Importantly, however, positive associations between stimulation and ECD have not been found in all studies. In particular, a cross‐sectional study of Ghanaian preschoolers (Wolf & McCoy, 2019) found that family stimulation predicted *lower* scores on multiple developmental domains of IDELA. The authors hypothesized that such a counterintuitive result could be due to the relatively superficial measure of stimulation (which captured the quantity but not the quality of interactions) or reverse causality caused by some sort of compensatory effect (i.e., parents stimulated children with lower skills more), but the cross‐sectional nature of their data prevented testing of the latter hypothesis.

While prior research generally shows positive associations between stimulation and ECD in LMICs, there are methodological and conceptual issues that limit our understanding of how stimulation might impact children's outcomes in these settings. First, most studies have relied on cross‐sectional data and conventional covariate adjustment (i.e., controlling for observed characteristics like socioeconomic status), which raises concerns about selection bias and reverse causality. Indeed, a bioecological perspective recognizes complex interactions between individual and contextual factors in child development (i.e., multifactorial causation), which suggests that conventional covariate adjustment typically does not solve issues of selection bias in developmental science, as some characteristics that covary with stimulation and ECD (i.e., confounders) are difficult to observe (e.g., genetic factors; Duncan et al., 2004; Gennetian et al., 2008). As such, it is possible that the existing literature has over‐stated the true effects of stimulation on children's developmental outcomes. Second, very few cross‐national investigations of early stimulation have considered the multidimensionality of ECD. Instead, most studies have relied on coarse measures of child outcomes, like the ECDI, that assess a limited range of skills and behaviors and do not provide sufficient precision to assess differences among domains. In contrast, studies from LMICs using more precise measures of ECD have focused on individual countries or settings, making it difficult to assess similarities and differences in links between stimulation and ECD across cultural settings. Finally, most research on stimulation in both LMICs and HICs has focused on maternal stimulation (e.g., Cuartas, Rey‐Guerra, et al., 2020), overlooking the likely importance of fathers and other adult caregivers and showing the need for further research with comprehensive conceptualizations of stimulation that consider all relevant adult caregivers.

### 1.3 The current study

This study aims to assess the links between family stimulation and young children's emergent numeracy, literacy, motor, social‐emotional, and executive function skills across five LMICs for which we identified comparable longitudinal data. Specifically, we use data from Bangladesh, Bhutan, Cambodia, Ethiopia, and Rwanda that include information on children's development assessed by the IDELA, a comprehensive, multi‐domain, direct assessment of ECD widely used in LMICs (Pisani et al., 2018; Wolf et al., 2017). We follow prior research from LMICs (e.g., Jeong et al., 2017; Wolf & McCoy, 2019) and operationalize stimulation as a unidimensional construct based on the primary caregiver's reports of whether or not adults in the household (i.e., mothers, fathers, and other caregivers) engaged with children in a set of basic stimulation activities (e.g., reading, playing, singing) in the prior week. Furthermore, we follow best‐practice recommendations for mitigating selection bias in observational developmental research (Duncan et al., 2004; Foster, 2010; Miller et al., 2016) and use conservative estimation methods and robustness checks to evaluate the internal validity of estimated relations between stimulation and ECD. Our hypothesis, informed by our conceptual model, is that children who are reported to receive more stimulation by their caregivers will exhibit better numeracy, literacy, social‐emotional, motor, and executive function skills. Considering quantitative research showing positive links between family stimulation and ECD and qualitative evidence indicating variation in how family stimulation manifests across cultures and settings, we hypothesized some variability in the associations between stimulation and ECD outcomes across studies.

## 2 METHODS

### 2.1 Data and participants

Data come from five longitudinal studies with two time points each: (1) Bangladesh Early Years Preschool Program (Spier et al., 2018), (2) Bhutan's National Early Childhood Care and Development Evaluation Study (Save the Children, 2015), (3) Cambodia First Read (Pisani et al., 2016), (4) Quasi‐experimental Longitudinal Study in Tigray Region in Ethiopia (Seiden et al., 2018), and (5) Rwanda's Advancing the Right to Read Program (Iwamoto et al., 2019). Each of these studies administered questionnaires for parents or children's main caregivers and direct assessments of children's skills using the IDELA (Pisani et al., 2018) at baseline (time 1; T1) and follow‐up (time 2; T2).

All studies were evaluations of different ECD programs (Bangladesh was experimental and the other four non‐experimental), ranging from early childhood care and education to integrated packages to promote young children's health and development. Sample sizes ranged from 382 (Cambodia) to 1856 children (Bangladesh). Children's mean age in months in T1 ranged from 49.3 (Bangladesh) to 58.8 (Cambodia). Finally, the time gap between T1 and T2 ranged from approximately 8 months (Bhutan) to 2 years (Cambodia and Ethiopia). Table 1 presents a summary of the five studies, and Supplemental materials Appendix A presents descriptions of each study's procedures, sample, and main results, and descriptive statistics for each study's sample (Tables S1–S5).

**TABLE 1:** Descriptions of the five studies.

	Early years preschool program (EYPP)	Early childhood care and development (ECCD) evaluation study	Cambodia first read	Quasi‐experimental longitudinal study	Advancing the right to read program (ARRP)
Country	Bangladesh	Bhutan	Cambodia	Ethiopia	Rwanda
Coverage	Meherpur district	National	Kampong Cham, Kratie, and Prey Veng	Tigray Region	Gasabo, Ngororero, Nyabihu, and Kicukiro
Sample size	1856	1377	382	693	596
Sample	Children from 100 schools in one district	Children from 120 ECCD centers across 9 districts and a comparison group not attending ECCD.	Children from 29 villages.	Children from five intervention woredas and three comparison woredas	Children attending 60 schools in four districts
Intervention	Center‐based program that provided training to pre‐primary teachers	Early childhood care and development services	Provision of age‐appropriate books, parenting supports, and community bonds	Early childhood care and development, basic education, school health and nutrition, and maternal and new‐born health	Supporting teachers and parents to effectively teach reading and writing
Design	Randomized controlled trial	Non‐experimental comparison	Non‐experimental comparison	Non‐experimental comparison	Non‐experimental comparison (difference in differences)
Findings	Positive impacts on children's numeracy, literacy and social‐emotional skills. No impact on parental stimulation	Associations between gains in IDELA scores and ECCD attendance, but no evidence for links between ECCD and parental stimulation	No significant associations between First Read and child outcomes or parental stimulation.	No results available to this date	Links between the ARRP and IDELA scores and parental stimulation.
Year of data collection					
T1	2018	2015 March	2016	2017	2018
T2	2019	2015 November	2018	2019	2019
Percent female	49	50	52	49	49
Mean (SD) age in months in T1	49.26 (3.44)	50.41 (8.52)	58.79 (11.03)	54.80(9.62)	53.23(6.51)
Mean (SD) IDELA score in T1	34% (18)	22% (15)	41% (19)	31% (16)	31% (13)
Mean (SD) stimulation in T1	5.36 (2.30)	6.59 (2.38)	5.24 (2.12)	5.72 (2.98)	4.04 (2.63)

### 2.2 Measures

#### 2.2.1 Child development

The IDELA is a direct assessment of preschool‐age children's skills that comprises 22 core subtasks, each containing multiple items. It was developed to be a holistic and easy‐to‐implement tool to measure the development of children ages 3.5–6.5 years in different national and cultural contexts. The typical assessment lasts about 30 min and requires minimal materials. To date, the IDELA is one of the few open‐access tools available to conduct valid direct assessments of children's development in LMICs (Fernald et al., 2017). The original design and studies examining its dimensionality and reliability indicate that the IDELA captures five domains of ECD: emergent numeracy, emergent literacy, motor, social‐emotional, and executive function (Pisani et al., 2018; Wolf et al., 2017). We confirmed the five‐domain factor and subtask structure using confirmatory factor analysis (CFA) on the pooled data with a maximum‐likelihood estimator, finding evidence of construct validity of the IDELA in each of the five studies considering conventional criteria for model fit (Hu & Bentler, 1999; for further details, see supplemental files Figures S3, S6, S9, S12, and S15).

While the IDELA was translated and subject to small adaptations to wording and stimulus material by local experts for the purpose of each study to ensure contextual relevance, each domain consisted of common sets of core subtasks and items (Table 2). Emergent numeracy was assessed using 39 items grouped into eight subtasks capturing skills in size and length discrimination, number identification, sorting abilities, shape identification, one‐to‐one correspondence, basic addition and subtraction, and simple problem solving. Emergent literacy was measured using 38 items grouped into six subtasks related to print awareness, letter identification, expressive vocabulary, emergent writing, sound discrimination, and listening comprehension. Social‐emotional skills were assessed with five subtasks (14 items) related to self‐awareness, emotion regulation, empathy, friendship, and problem solving. The motor domain comprised four subtasks, with nine items in total, to assess gross and fine motor skills. Two subtasks, one on short‐term memory and other on inhibitory control, comprised the executive‐function domain (9 items in total). We followed the developers’ standard scoring procedures to calculate the percentage of correct items in each domain (Pisani et al., 2018; see Appendix B Figures S3, S6, S9, S12, and S15 for information on reliability).

**TABLE 2:** IDELA subtasks by domain.

Emergent numeracy	Emergent literacy	Motor development	Social‐emotional skills	Executive function
**• Basic addition and subtraction**	• Emergent writing	• Hopping on one foot	• Self‐awareness	• Short‐term memory
**• Size and length discrimination**	• Print awareness	• Copying a shape	• Emotional awareness and regulation	• Inhibitory control
**• Sorting abilities**	• Listening comprehension	• Drawing a human figure	• Empathy	
**• Shape identification**	• Expressive vocabulary	• Folding paper	• Peer relations	
**• Number identification**	• Letter identification		• Conflict resolution and problem solving	
**• One‐to‐one correspondence**	• Sound discrimination			
**• Simple problem solving**				

#### 2.2.2 Stimulation

In each study, caregivers reported whether (yes/no) mothers, fathers, and or other caregivers engaged with the child in nine stimulation activities in the week prior to the survey, including (1) reading books or looking at picture books, (2) telling stories, (3) singing songs to or with the children, including lullabies, (4) taking children outside of the homes, for example, to markets or to visit relatives, (5) playing simple games, (6) naming objects or drawing things, (7) teaching something new to the children, like new words or how to do something, (8) teaching the alphabet or letters to the children, and (9) playing counting games or teaching numbers to the children. These items were adapted from the Indicators of Family Care for Development (Kariger et al., 2013), which were developed for implementation in multi‐country surveys, particularly those in LMICs. Consistent with extensive prior research from LMICs (e.g., Cuartas, Rey‐Guerra et al., 2020; Frongillo et al., 2017; Jeong et al., 2017; McCoy et al., 2018; Rey‐Guerra et al., 2022; Wolf & McCoy, 2019), we found strong evidence for a one‐factor solution according to exploratory factor analysis (i.e., eigenvalues) and CFA (i.e., adequate model fit, considering a Root Mean Square Error of Approximation <0.08, Tucker Lewis Index >0.9, and Comparative Fit Index >0.9; Hu & Bentler, 1999) using the pooled data and a maximum‐likelihood estimator (for further details, see Appendix B Figures S1‐S2, S4‐S5, S7‐S8, S10‐S11, S13‐S14). Therefore, we computed a unidimensional index of caregiver‐child engagement across all stimulation activities, summing the total number of activities children engaged in with any adult caregiver in the week prior the survey (possible range = 0 − 9).

#### 2.2.3 Covariates

We used demographic and contextual information collected through the caregiver surveys as covariates in the analytic models. First, caregivers reported children's age (in months), sex (male or female), maternal highest levels of schooling (none, primary, secondary, higher, or non‐formal education [e.g., adult education]), and mothers’ age, which we grouped into three categories that were used in the original survey of Cambodia First Read study (18–24, 25–35, +36 years). Second, following the Indicators of Family Care for Development, caregivers were asked about the availability of different types of books (e.g., storybooks, textbooks, newspapers) and toys (e.g., homemade, store‐bought, household objects used as toys, drawing materials) in the households. We used this information to compute the total number of books (possible range = 0 − 5) and toys (possible range = 0 − 9) to characterize the availability of play or learning materials in the home. Finally, the caregiver surveys asked about different basic dwelling characteristics (e.g., access to electricity, roof materials) and household assets (e.g., television, fridge) that were relevant for the specific contexts of each study (see Appendix C (Table S6) for details). Using these data, we computed country‐specific household‐wealth indices using polychoric principal‐component analysis, following the algorithm presented by Filmer and Pritchett (2001). The first component, which represented household wealth, accounted for between 39% (in Rwanda) and 49% (in Bangladesh) of total variance in the sample. Finally, each dataset contained an indicator for “treatment status” (e.g., random assignment to treatment or control group in Bangladesh; participating or not in ECCD in Bhutan; see Appendix A for further details on the intervention evaluated in each study), which we used as an additional covariate in our models to account for potential intervention effects.

### 2.3 Analysis

We conducted pooled and country‐specific analyses of the associations between stimulation and each domain of child development using two analytic approaches that leverage between‐child and within‐child variation. Pooled analyses included country fixed effects to compare exclusively children living in the same country, taking into account the evidence for potential non‐invariance of the IDELA across selected LMICs (Halpin et al., 2019). We present both unstandardized results that reflect variables in their original metric (e.g., percentage correct in IDELA, number of activities for stimulation) and standardized results that reflect predictor and outcome variables that were z‐scored within countries to have means of zero and standard deviations of one. All regression models included heteroskedasticity‐robust standard errors. We followed best‐practice recommendations for handling missing data and used multiple imputation by chained equations (MICE; Royston & White, 2011; White et al., 2011), imputing 20 datasets (for further information on missing cases, see Tables S1–S5). We conducted all analyses in Stata 17.0.

#### 2.3.1 Between‐child variation: random‐effects (RE) models

Our first methodological approach follows prior cross‐sectional studies and exploits between‐child variation with a RE model. In particular, we estimated the model presented in Equation (1):

(1) $$\;Y_{it}=\;\lambda +\beta Stimulation_{it}+C_{i}\gamma +V_{it}\tau +\omega_{i}+\mu_{it}$$

In this model, *Y_it_* represented the outcome variable (e.g., emergent numeracy, social‐emotional skills) for child *i* at time *t*, *Stimulation_it_* was the stimulation index, *C_i_* was a vector of time‐invariant characteristics (i.e., children's sex, maternal education and age, household wealth, treatment status), *V_it_* was a vector of time‐varying characteristics (i.e., availability of toys and books), ω_*i*_ was a set of country fixed effects (i.e., a binary indicator for each country), and μ_*it*_ represented variation left unexplained. In the model, β represented the coefficient of interest for the association between stimulation and ECD outcomes.

The RE models’ validity for interpreting β as “causal” relies on the assumption that after controlling for a set of covariates, there are no confounders left unexplained in the model (i.e., in μ_*it*_). This is a strong and likely implausible assumption, given multifactorial causation of child development and the likely omission of unobserved differences among children that correlate with their stimulation and ECD outcomes (e.g., genetic makeup).

#### 2.3.2 Within‐child variation: Child fixed effects (FE) models

The second methodological approach follows best‐practice recommendations for mitigating issues of selection bias in observational developmental studies (Foster, 2010; Miller et al., 2016) and leverages within‐child variation with child fixed effect (FE) models. Child FE models overcome some limitations of RE models by using each child as his or her own “control” within the longitudinal design, thus controlling for all time‐invariant characteristics (e.g., genetic makeup) and mitigating issues of selection due to differences among children (Angrist & Pischke, 2011; Miller et al., 2016). Specifically, we estimated the model presented in Equation 2.

(2) $$\;Y_{it}=\;\beta Stimulation_{it}+V_{it}\tau +\pi_{i}+\varepsilon_{it}$$

In this model, π_*i*_ represented the FEs or, simply stated, unique regression intercepts for each child, and ε_*it*_ was residual variation. β represented the association between stimulation and ECD outcomes after accounting for a set of time‐varying characteristics (*V_it_*) and all observed and unobserved time‐invariant confounders (using the FEs). Although the FE model discards concerns about selection due to unobserved time‐invariant confounders, the validity of this model for estimating causal effects still relies on the (more plausible) assumption that there are not unobserved time‐varying confounders that should be in the model.

## 3 RESULTS

Table 3 summarizes unstandardized results from the pooled‐RE models (Table S7 presents results for all covariates and Table S9 for models with standardized variables). In general, higher levels of caregiver‐reported stimulation were associated with better ECD outcomes as measured by the IDELA, even after accounting for children's sex, age, mothers’ age and education, household wealth, availability of learning materials, treatment status, and country FEs. Specifically, caregivers’ engagement in one additional activity with children predicted 0.70 percentage points increments in correct answers in the numeracy domain (*SE* = 0.10; *p* < 0.001), 0.75 in literacy (*SE* = 0.11; *p* < 0.001), 0.76 in social‐emotional (*SE* = 0.11; *p* < 0.001), 0.71 in motor (*SE* = 0.13; *p* < 0.001), and 0.58 percentage points in executive function (*SE* = 0.13; *p* < 0.001). In standardized units, these results indicate that a one *SD* increment in family stimulation relates to increments between 0.05 *SD* (in EF) to 0.08 *SD* (in numeracy, literacy, and social‐emotional skills).

**TABLE 3:** Summary of random‐effects models exploiting between‐child variation to estimate the association between family stimulation and children's ECD outcomes using pooled data from five studies.

	Numeracy	Literacy	Social‐emotional	Motor	Executive Function
Stimulation	0.70***	0.75***	0.76***	0.71***	0.58***
	(0.10)	(0.11)	(0.11)	(0.13)	(0.13)
Covariates	Yes	Yes	Yes	Yes	Yes
Country FEs	Yes	Yes	Yes	Yes	Yes
Child FEs	No	No	No	No	No
Observations	9808	9808	9808	9808	9808
Number of children	4904	4904	4904	4904	4904

*Notes*: Robust standard errors in parentheses. Covariates: children's sex and age, mothers’ age and education, household wealth, availability of toys and books, treatment status. See Table S7 for full results and Table S9 for standardized coefficients.

^+^ *p* < 0.1, *p < 0.05, **p < 0.01, ***p < 0.001.

The positive associations between family stimulation and children's ECD outcomes were also robust to a more conservative child FE approach, with slightly larger estimates (see Table 4 for summary, Table S8 for full results, and Table S10 for results using standardized variables). Specifically, caregivers’ engagement in one additional activity with children predicted 0.80 percentage‐points increments in correct answers in the numeracy domain (*SE* = 0.16; *p* < 0.001), 0.87 in literacy (*SE* = 0.17; *p* < 0.001), 1.07 in social‐emotional (*SE* = 0.17; *p* < 0.001), 0.83 in motor (*SE* = 0.20; *p* < 0.001), and 0.79 percentage points in executive function (*SE* = 0.19; *p* < 0.001). In standardized units, the associations were larger for the social‐emotional domain (0.11 *SD*), followed by numeracy and literacy (0.09 *SD*), and smaller for motor and executive function (0.07 *SD*).

**TABLE 4:** Summary of child fixed‐effects models exploiting within‐child variation to estimate the association between family stimulation and children's ECD outcomes using pooled data from five studies.

	Numeracy	Literacy	Social‐emotional	Motor	Executive function
Stimulation	0.80***	0.87***	1.07***	0.83***	0.79***
	(0.16)	(0.17)	(0.17)	(0.20)	(0.19)
Covariates	Yes	Yes	Yes	Yes	Yes
Child FE	Yes	Yes	Yes	Yes	Yes
Observations	9808	9808	9808	9808	9808
Number of children	4904	4904	4904	4904	4904

*Notes*: Robust standard errors in parentheses. Covariates: availability of books and toys. See Table S8 for full results and Table S10 for standardized coefficients.

^+^ *p* < 0.1, *p < 0.05, **p < 0.01, ***p < 0.001.

**FIGURE 1:**
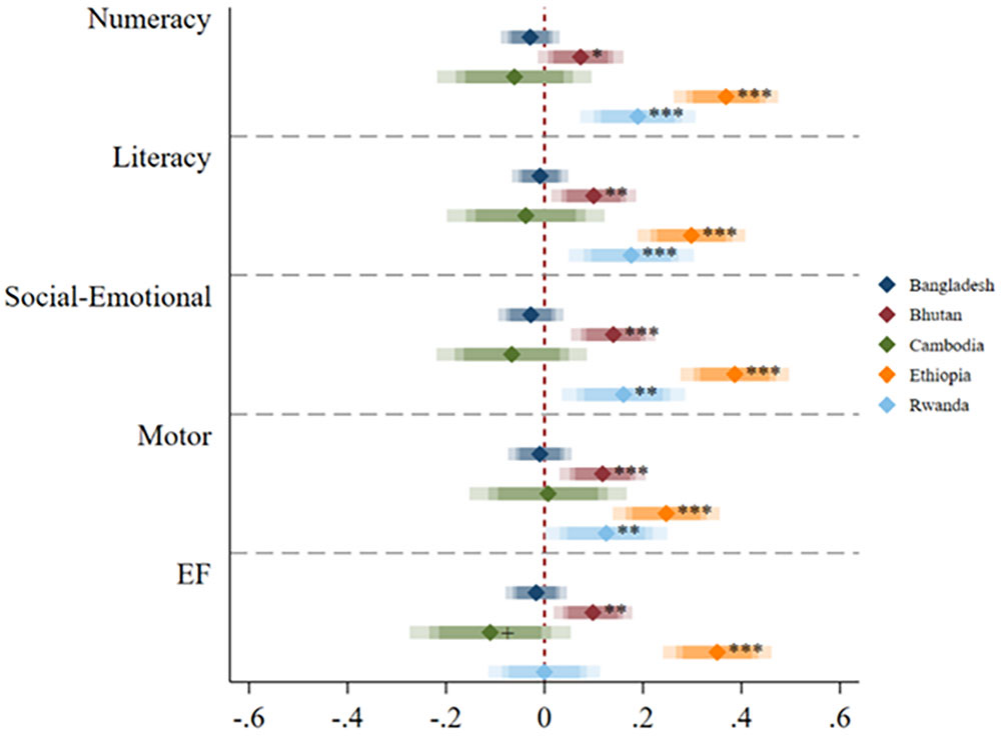
Summary of the standardized associations between family stimulation and children's ECD outcomes using child FE models, by country. *Note*: Shaded bars (from lighter to darker color) represent 90%, 95%, and 99% CI. ^+^ *p* < 0.1, ^*^ *p* < 0.05, ^**^ *p* < 0.01, ^***^ *p* < 0.001.

Results from separate models using child FE within each study show differences in the associations between stimulation and children's ECD outcomes across countries (Figure 1 and Tables S11–S15). In particular, the links between stimulation and all developmental domains were stronger in Ethiopia relative to other countries, with standardized associations between 0.25 *SD* (for motor skills) and 0.39 *SD* (for social‐emotional skills). The associations between stimulation and ECD outcomes were also statistically significant and positive in Rwanda and Bhutan, ranging from 0.07 *SD* (for numeracy in Bhutan) to 0.19 *SD* (for numeracy in Rwanda). Importantly, however, we did not find any statistically significant positive associations between stimulation and children's ECD outcomes in Bangladesh or Cambodia. Indeed, we actually observed one marginally significant negative association between stimulation and children's executive function in Cambodia (β = − 0.11; *SE* = 0.06; *p* < 0.1).

## 4 DISCUSSION

Global initiatives like the SDGs and the Nurturing Care Framework (Britto et al., 2017) have highlighted the urgency of promoting early stimulation as a promising means for supporting young children around the world. Despite these efforts and prior studies showing associations between family stimulation and positive ECD outcomes (Cabrera et al., 2020; Jeong et al., 2017), most evidence on stimulation comes from HICs, relies on cross‐sectional data and correlational analysis, and/or employs measures of ECD that were reported by parents and limited in scope. Therefore, there is a need for more research examining the links between family stimulation and ECD outcomes using more internally valid designs with longitudinal data and more precise measures of ECD to inform generalizable theories, advocacy efforts, and global policies.

To address these gaps, the present study examined the links between family stimulation and young children's directly assessed ECD outcomes using longitudinal data from Bangladesh, Bhutan, Cambodia, Ethiopia, and Rwanda. Our findings revealed significant positive links between family stimulation and ECD outcomes. In particular, children who received additional sources of stimulation from their mothers, fathers, or other caregivers—including activities like reading, counting, and playing, were found to score on average higher in their early numeracy, literacy, social‐emotional, motor, and executive function skills. Effect sizes for these differences were somewhat small, with differences in outcomes ranging from approximately 0.05 to 0.11 *SD* per one *SD* increment in activities received. Nevertheless, the magnitudes of these estimates were consistent with prior research; for example, a cross‐sectional national study in Colombia found standardized associations between family stimulation and IDELA domains ranging between 0.05 and 0.08 *SD*. These results were also consistent with other studies examining the links between stimulation and coarser unidimensional measures of ECD in LMICs (Alam et al., 2021; Cuartas, Rey‐Guerra et al., 2020; Duc, 2016; Jeong et al., 2017) and randomized controlled trials of parenting interventions in LMICs, which showed effects on caregivers’ engagement in stimulation activities and young children's cognitive, language, social‐emotional, and motor skills (Jeong et al., 2021), probably suggesting that stimulation is a mechanism explaining the observed impacts on ECD outcomes.

Measurement models indicate that the nine stimulation activities appear to load into a unidimensional family stimulation construct and the findings from models using pooled data showed that this unidimensional index of family stimulation predicts different domains of ECD somewhat equivalently. This is consistent with theory and evidence indicating that family engagement in enriching activities that provide opportunities for movement behaviors, exploration, communication, modeling, scaffolding, problem‐solving, and emotional responsiveness (among others) in a playful environment might be beneficial for the development of cognitive, social‐emotional, and motor skills (Bandura, 1986; Black et al., 2017; Vygotsky, 1981). While some researchers have tried to distinguish between “cognitive” and “social‐emotional” stimulation (Bornstein & Putnick, 2012), all the stimulation activities examined in this study load into a single unidimensional construct that predicts increments in numeracy, literacy, social‐emotional, motor, and executive function skills. These results collectively highlight the ways in which the activities that family members do with children can be supportive of multiple developmental domains, and contradicts the idea that stimulation is necessarily just cognitive or just social‐emotional in nature.

Furthermore, we observed differences in the associations between family stimulation and ECD outcomes across studies, with studies from Bangladesh and Cambodia exhibiting null associations. Based on our data, we cannot explain why this variability is occurring. At first glance, these results might contradict the idea that family stimulation is universally beneficial for children. Yet, it is possible that the nine relatively basic indicators used to measure family stimulation in these studies may be insufficient or inappropriate for capturing culturally specific behaviors and mechanisms through which parents and other caregivers in these settings support early childhood development (LeVine et al., 2008; Mesman et al., 2018; Morelli et al., 2017; Rogoff et al., 1993). Indeed, ethnographic evidence has shown differences in daily interactions, the ways the same activities (e.g., play) might manifest across cultures and settings (e.g., more face‐to‐face vs. more skin‐to‐skin contact, stimulation following child‐led play vs. amid household chores and work), and the amount and type of participation of children in peer play with relatives and or other peers of different ages (Cheung et al., 2021; Gaskins, 2015; Keller, 2003; Keller & Otto, 2009; Nag et al., 2019; Rogoff et al., 2017). These and other subtle differences in specific interactions and the manifestations of the same stimulation activities, which we cannot characterize in the current study, might well be relevant to explain the observed differences in estimates. Therefore, these findings stress the need for more culturally nuanced evidence and measurement approaches to clarify the precise forms of family stimulation that may contribute to ECD outcomes in LMICs, where the majority of young children live. At the same time, the studies from Bangladesh and Cambodia are not representative, therefore it is unknown whether the same null findings would hold for representative samples. Finally, the Cambodian sample comprised a slightly older cohort of children, but there are no theoretical reasons to think that such a difference could be important in explaining the null results.

### 4.1 Implications for policy and practice

The current study found that caregivers’ engagement in a set of basic stimulation activities with young children is associated with more positive development of children's early cognitive, social‐emotional, and motor skills in the majority—but not all—of the five LMICs studies. These findings, along with prior evidence showing generally low levels of family stimulation in LMICs (Cuartas, Jeong et al., 2020; Lu et al., 2020) and the importance of foundational skills for lifelong development, learning, and wellbeing (Berens & Nelson, 2019; Black et al., 2017; Trude et al., 2021), suggest the need for additional strategies aimed at promoting early stimulation to catalyze positive developmental trajectories. At the same time, heterogeneity in social norms, attitudes toward play, and manifestations of family stimulation across cultures and settings clearly demonstrate the need for further studies, specifically ethnographic and qualitative in nature, to elucidate the specific activities that might be acceptable, feasible, aligned with socialization goals, and promotive of children's ECD outcomes that are valued and needed within each context.

Parenting programs offer a direct platform to promote family stimulation of young children. A global systematic review and meta‐analysis of the literature that included 102 unique randomized controlled trials conducted in 14 HICs and 19 LMICs (Jeong et al., 2021), found that parenting interventions led to sizable improvements, on average, in parenting practices (*d* = 0.33) and parent‐child interactions (*d* = 0.39), as well as gains in children's cognitive (*d* = 0.32), language (*d* = 0.28), motor (*d* = 0.24), and social‐emotional development (*d* = 0.19), with greater effects in LMICs compared to HICs. Parenting programs have been implemented through home visits, group sessions, and in clinic appointments with varied duration and intensity (Aboud & Yousafzai, 2015), and there is limited evidence of differential effectiveness based on their duration, delivery approach, or setting (Jeong et al., 2021). Similarly, parenting programs have been effectively adapted to and implemented in multiple sociocultural contexts (Britto et al., 2017; Gardner, 2017; Grantham‐McGregor et al., 2020; McCoy et al., 2020; Scott & Gardner, 2015). The effectiveness and flexibility of parenting programs makes them a promising strategy to promote early stimulation at scale. Given evidence from this study and others (e.g., Cuartas, Jeong et al., 2020) regarding the important role played by multiple caregivers in supporting stimulation, it will be important to engage fathers and other caregivers in these programs, as existing interventions have largely focused on mothers (Aboud & Yousafzai, 2015).

Furthermore, just as children's development takes place within complex ecological systems (Bronfenbrenner & Morris, 2007), proximal and distal factors shape caregiver‐child interactions by affecting who is interacting with young children and under what conditions. Economic and social conditions influence caregivers’ mental health and shape their ability to spend time with their young children, and the quality of such interactions (Britto et al., 2017). Moving forward, more efforts are needed to put in place policies that enable caregivers to spend high‐quality time with their children, such as income supplements (Lombardi, 2021), supportive employment conditions including paid‐maternal‐and‐parental leaves (Heymann et al., 2013; O'Brien, 2009), and mental‐health supports (Britto et al., 2017), among others.

### 4.2 Limitations and future directions

The current study has limitations that inform future directions for research. First, the measure of family stimulation used in this study was reported by the main caregivers, usually the mothers, raising concerns about measurement bias and underreporting of other caregivers’ stimulation if the main caregivers were not aware of such interactions. The measure also focused on behaviors that took place the week before the survey, which might help regarding recall bias but might be sensitive to variations in family routines or the specific timing when the interviews were conducted. Furthermore, the measure of stimulation used across these studies did not include details regarding the quality, frequency, or other characteristics (e.g., child‐led vs. adult‐directed) of the stimulation interactions, nor regarding culturally specific practices. Future research should employ more detailed measures of stimulation, using mothers’, fathers’, and other caregivers’ reports, gathering information on the characteristics of the stimulation, and employing direct assessments of the interactions. Second, while the stimulation index exhibited adequate psychometric properties according to conventional criteria (Hu & Bentler, 1999), it is difficult to assess how caregivers defined and understood each independent activity included in the index (e.g., playing). Therefore, it is difficult to assess whether differences in observed associations across studies are due to differential understanding of the stimulation items. Future studies should explore local conceptualizations and manifestations of stimulation and identify valued activities or ways in which activities are conducted to collect in future data‐collection efforts and study in future research.

Third, despite the importance of siblings and peers in engaging with young children in stimulation activities without adult supervision (Rogoff et al., 1993), the current study had no specific information on child‐to‐child (e.g., siblings, peers) stimulation or about stimulation outside of household environments. Similarly, we did not have information about children's stimulation in early childhood‐care and educational settings (e.g., preschool, daycare). Future studies should analyze the independent, supplementary, and complementary effects of stimulation by different caregivers in different settings, complementing recent findings from a longitudinal study in the United States (Cabrera et al., 2020) and a cross‐sectional study in Colombia (Rey‐Guerra et al., 2022) that identified positive links between both family and center‐based stimulation and ECD outcomes.

Fourth, while the study makes important contributions by employing a more internally valid methodological approach (child FE) with data from five LMICs, there are potential threats to validity that limit the inferences that can be drawn from this study. On the one hand, child FE relies on the assumption that no relevant time‐varying confounders are uncontrolled in the model (Angrist & Pischke, 2011). Therefore, there could be potential biases in estimates of causal effects introduced by unobserved time‐varying confounders (e.g., caregiver depression, exposure to violence, food insecurity, or access to social programs). While it is impossible to discard all potential confounders, the current study complements extensive prior research (e.g., Cabrera et al., 2020; Jeong et al., 2017; Jeong et al., 2016) by providing more internally valid evidence about positive links between family stimulation and ECD across multiple settings. On the other hand, the present study relied on data from five studies that are not nationally representative. Consequently, these findings have limited external validity. Future research should complement this study by leveraging longitudinal designs, testing the robustness of findings to alternative identifying assumptions, and conducting replications in more LMICs using nationally representative samples.

Fifth, there is some variation in children's age and in the gaps between T1 and T2 between studies, ranging from approximately eight months to two years. Our models partially accounted for such variation by controlling for child age (in RE models) and for time‐invariant characteristics (in FE models). While there are no apparent trends in the estimated coefficients according to the gaps between T1 and T2, the current data make it difficult to assess the extent to which associations between family stimulation and ECD varies in different developmental periods (as we did not have the exact date when each assessment was conducted). Theoretically, such gaps could have significant developmental implications, as the effects of stimulation could be stronger early in life when the brain is more malleable in response to stimuli and the effects of early experiences might accumulate as children grew older (Berens & Nelson, 2019; Cunha & Heckman, 2007). Therefore, future studies should further explore issues of timing in the links between stimulation and ECD.

## 5 CONCLUSION

We found links between family stimulation, as measured by engagement in nine basic activities, and the development of young children's early numeracy, literacy, social‐emotional, motor, and executive function skills in studies from five LMICs. Yet, we observed sizable variability in such links across studies. These findings suggest the need for additional research aimed at understanding culturally specific behaviors and mechanisms through which caregivers may support early childhood development and highlight the importance of policies and programs aimed at promoting family stimulation to catalyze positive developmental trajectories in LMICs.

## CONFLICT OF INTEREST STATEMENT

The authors do not have any conflict of interest.

## Supporting information

Supporting Information

## Data Availability

Data used in this study can be requested to Save the Children.
