# Supplementary material for: Family play, reading, and other stimulation and early childhood development in five low‐and‐middle‐income countries
Source: Dev Sci. 2023 Apr 28;26(6):e13404. doi: 10.1111/desc.13404 (PMC11475363; doi:10.1111/desc.13404)
Supplement: Supplementary file 1 — Supporting Information [file DESC-26-e13404-s001.docx]

**Family Play, Reading, and Other Stimulation and Early Childhood Development in Five Low-and-Middle-Income Countries**

**Supplemental files**

Contents

[Appendix A: Description of the Studies 2](#_Toc89074122)

[Bangladesh Early Years Preschool Program 2](#_Toc89074123)

[Bhutan’s National Early Childhood Care and Development Evaluation Study 4](#_Toc89074124)

[Cambodia’s First Read Program 6](#_Toc89074125)

[Quasi-experimental Longitudinal Study in Tigray Region in Ethiopia 8](#_Toc89074126)

[Rwanda’s Advancing the Right to Read Program 10](#_Toc89074127)

[Appendix B: Psychometric analysis 12](#_Toc89074128)

[Bangladesh 12](#_Toc89074129)

[Bhutan 15](#_Toc89074130)

[Cambodia 18](#_Toc89074131)

[Ethiopia 20](#_Toc89074132)

[Rwanda 24](#_Toc89074133)

[Appendix C. Wealth index 27](#_Toc89074134)

[Appendix D. Full results 28](#_Toc89074135)

[Appendix E. Results for models with standardized variables 30](#_Toc89074136)

[Appendix F. Results for country-specific models 32](#_Toc89074137)

# Appendix A: Description of the Studies

## Bangladesh Early Years Preschool Program

The Early Years Preschool Program Impact Evaluation (Spier et al., 2018) was an experimental impact evaluation (i.e., randomized controlled trial) of a center-based program that provided five sessions of training and ongoing support (with bi-monthly refresher trainings, for a total of 4 sessions) to pre-primary teachers. The program also included one day of training for teachers on how to support parents to engage in activities that might promote young children’s mathematics and literacy skills.

A total of 100 schools in the Meherpur district participated in the study. Half were randomly selected to the treatment condition (i.e., receiving the Early Years Preschool Program) and half to a control condition (i.e., receiving pre-primary education as usual). A random sample of about 20 children in each study setting was selected. A total of 32 data collectors and four field supervisors were trained for about one week on the procedures and measurement instruments, particularly the IDELA. All data were collected in participants’ households in 2018 (baseline or T1) and 2019 (follow-up or T2).

The impact evaluation of the Early Years Preschool Program demonstrated statistically significant impacts on children’s literacy (0.23 SD), numeracy (0.30 SD), and social-emotional skills (0.34 SD). The program had no effects on parental stimulation.

Table S1: Descriptive statistics for Bangladesh

| Variable | T1 | | | T2 | | | T2-T1 |
| --- | --- | --- | --- | --- | --- | --- | --- |
|  | N | α | M(SD) or % | N | α | M(SD) or % | *p-value* |
| **Child** |  |  |  |  |  |  |  |
| Age in months | 1856 |  | 49.26 | 1685 |  | 62.28 | <0.001 |
|  |  |  | (3.44) |  |  | (3.44) |  |
| Sex (female = 1) | 1856 |  | 49% | 1685 |  | 49% |  |
|  |  |  | (50) |  |  | (50) |  |
| **Child Development** |  |  |  |  |  |  |  |
| IDELA (overall) | 1856 | .87 | 34% | 1685 | .86 | 60% | <0.001 |
|  |  |  | (18) |  |  | (18) |  |
| Socio-Emotional | 1856 | .62 | 28% | 1814 | .70 | 51% | <0.001 |
|  |  |  | (18) |  |  | (23) |  |
| Emergent Literacy | 1856 | .79 | 29% | 1814 | .83 | 55% | <0.001 |
|  |  |  | (19) |  |  | (25) |  |
| Emergent Numeracy | 1856 | .77 | 35% | 1814 | .81 | 54% | <0.001 |
|  |  |  | (18) |  |  | (21) |  |
| Motor | 1856 | .75 | 42% | 1685 | .70 | 73% | <0.001 |
|  |  |  | (28) |  |  | (23) |  |
| Executive Function | 1856 | .42 | 36% | 1814 | .55 | 55% | <0.001 |
|  |  |  | (22) |  |  | (26) |  |
| **Caregiver** |  |  |  |  |  |  |  |
|  |  |  |  |  |  |  |  |
| *Age* | 1856 |  |  | 1685 |  |  |  |
| <18 - 24 | N/A |  | 32% | N/A |  | 26% |  |
|  |  |  |  |  |  |  |  |
| 25-35 | N/A |  | 59% | N/A |  | 58% |  |
|  |  |  |  |  |  |  |  |
| >36 | N/A |  | 9% | N/A |  | 17% |  |
|  |  |  |  |  |  |  |  |
| *Educational Level* | 1850 |  |  | 1087 |  |  |  |
| None | N/A |  | 12% | N/A |  | 12% |  |
|  |  |  |  |  |  |  |  |
| Primary | N/A |  | 23% | N/A |  | 23% |  |
|  |  |  |  |  |  |  |  |
| Secondary | N/A |  | 56% | N/A |  | 56% |  |
|  |  |  |  |  |  |  |  |
| Higher | N/A |  | 9% | N/A |  | 9% |  |
|  |  |  |  |  |  |  |  |
| *Stimulation* |  |  |  |  |  |  |  |
| Parental stimulation | 1856 | .71 | 5.36 | 1814 | .70 | 6.13 | <0.001 |
|  |  |  | (2.30) |  |  | (2.17) |  |
| **Context** |  |  |  |  |  |  |  |
| Wealth | 1856 |  | 0.00 | N/A |  |  |  |
|  |  |  | (0.84) |  |  |  |  |
| Toys | 1856 | .40 | 5.00 | 1813 | .42 | 6.36 | <0.001 |
|  |  |  | (1.43) |  |  | (1.43) |  |
| Books | 1856 | .54 | 2.14 | 1813 | .52 | 3.04 | <0.001 |
|  |  |  | (1.05) |  |  | (0.98) |  |

## Bhutan’s National Early Childhood Care and Development Evaluation Study

The Bhutan’s National Early Childhood Care and Development Evaluation (ECCD) Study (Pisani et al., 2017) was an observational (i.e., non-experimental) evaluation of ECCD services in Bhutan. The study used the National Statistics Bureau's regional classification of districts to select a representative sample of ECCD centers. A random sample of 10–15 children was selected in each center. Twenty-four enumerators with prior data-collection experience were selected to conduct data collection. The enumerators were trained for about six days on the measurement instruments and techniques for interviewing young children, among other topics. The study also included a comparison group of children who did not participate in any ECCD center-based programming. Child assessments took place in ECCD centers when possible and in central locations in village when children were not enrolled in ECCD. The data were collected in March 2015, at the beginning of the school year (baseline or T1), and November 2015, at the end of the school year (follow-up or T2).

Results from the evaluation revealed that having access to ECCD was associated with larger gains in multiple domains of early development. No associations were assessed between ECCD and parental stimulation.

Table S2: Descriptive statistics for Bhutan

| Variable | T1 | | | T2 | | | T2-T1 |
| --- | --- | --- | --- | --- | --- | --- | --- |
|  | N | α | M(SD) or % | N | α | M(SD) or % | *p-value* |
| **Child** |  |  |  |  |  |  |  |
| Age in months | 1347 |  | 50.41 | 1163 |  | 57.55 | <0.001 |
|  |  |  | (8.52) |  |  | (8.77) |  |
| Sex (female = 1) | 1377 |  | 50% | 1189 |  | 50% |  |
|  |  |  | (50) |  |  | (50) |  |
| **Child Development** |  |  |  |  |  |  |  |
| IDELA (overall) | 1377 | .85 | 22% | 1189 | .87 | 45% | <0.001 |
|  |  |  | (15) |  |  | (19) |  |
| Socio-Emotional | 1377 | .73 | 21% | 1189 | .72 | 40% | <0.001 |
|  |  |  | (17) |  |  | (21) |  |
| Emergent Literacy | 1377 | .66 | 15% | 1189 | .72 | 36% | <0.001 |
|  |  |  | (14) |  |  | (21) |  |
| Emergent Numeracy | 1377 | .75 | 25% | 1189 | .74 | 44% | <0.001 |
|  |  |  | (17) |  |  | (19) |  |
| Motor | 1377 | .75 | 24% | 1186 | .76 | 57% | <0.001 |
|  |  |  | (25) |  |  | (28) |  |
| Executive Function | 1377 | .58 | 33% | 1189 | .55 | 54% | <0.001 |
|  |  |  | (28) |  |  | (29) |  |
| **Caregiver** |  |  |  |  |  |  |  |
|  |  |  |  |  |  |  |  |
| *Age* | 1355 |  |  | 1171 |  |  |  |
| <18 – 24 | N/A |  | 18% | N/A |  | 11% |  |
|  |  |  |  |  |  |  |  |
| 25-35 | N/A |  | 66% | N/A |  | 69% |  |
|  |  |  |  |  |  |  |  |
| >36 | N/A |  | 15% | N/A |  | 20% |  |
|  |  |  |  |  |  |  |  |
| *Educational Level* | 1376 |  |  | 1189 |  |  |  |
| None | N/A |  | 35% | N/A |  | 38% |  |
|  |  |  |  |  |  |  |  |
| Primary | N/A |  | 12% | N/A |  | 12% |  |
|  |  |  |  |  |  |  |  |
| Secondary | N/A |  | 21% | N/A |  | 21% |  |
|  |  |  |  |  |  |  |  |
| Higher | N/A |  | 10% | N/A |  | 10% |  |
|  |  |  |  |  |  |  |  |
| *Stimulation* |  |  |  |  |  |  |  |
| Parental stimulation | 1377 | .79 | 6.59 | 1189 | .81 | 7.18 | <0.001 |
|  |  |  | (2.38) |  |  | (2.23) |  |
| **Context** |  |  |  |  |  |  |  |
| Wealth | 1370 |  | -0.02 | N/A |  |  |  |
|  |  |  | (1.59) |  |  |  |  |
| Toys | 1377 | .64 | 4.58 | 1189 | .66 | 5.63 | <0.001 |
|  |  |  | (2.00) |  |  | (2.01) |  |
| Books | 1377 | .75 | 2.14 | 1189 | .78 | 2.60 | <0.001 |
|  |  |  | (1.60) |  |  | (1.65) |  |
|  |  |  |  |  |  |  |  |

## Cambodia’s First Read Program

This was an observational (i.e., non-experimental) study that assessed change over time in IDELA scores and other outcomes for children who participated in First Read (Pisani, Dib, & Khoy, 2016). First Read aimed to support poor and marginalized children under six in three provinces in Cambodia to learn and develop their full potential by developing and providing age-appropriate, high-quality books (partnering with NGOs and other actors), conducting parenting sessions to promote early stimulation, and promoting community participation to support local structures and strengthen the local capacity.

The study included 29 villages in Kampong Cham, Kratie, and Prey Veng provinces. Children were randomly sampled from the project villages. All villages included in the study received First Read for some time (i.e., Phase 1), but for the comparison group First Read was phased-out for Phase 2 whereas for the intervention group First Read continued in the second Phase II. The researchers collected both quantitative and qualitative data. Enumerators were trained during four days on how to administer the measurement instruments and three Save the Children leaders supervised data collection. Data were collected in participants’ households in 2016 (baseline or T1) and 2018 (follow-up or T2).

The results of the study showed no significant association between attending First Read sessions and child-development (IDELA) outcomes, but there were statistically significant associations between the intervention and book ownership. There was no evidence of links between First Read and parental stimulation.

Table S3: Descriptive statistics for Cambodia

| Variable | T1 | | | T2 | | | T2-T1 |
| --- | --- | --- | --- | --- | --- | --- | --- |
|  | N | α | M(SD) or % | N | α | M(SD) or % | p-value |
| **Child** |  |  |  |  |  |  |  |
| Age in months | 382 |  | 58.79 | 353 |  | 65.79 | <0.001 |
|  |  |  | (11.03) |  |  | (11.03) |  |
| Sex (female = 1) | 353 |  | 52% | 353 |  | 52% |  |
|  |  |  | (50) |  |  | (50) |  |
| **Child Development** |  |  |  |  |  |  |  |
| IDELA (overall) | 382 | .86 | 41% | 353 | .77 | 74% | <0.001 |
|  |  |  | (19) |  |  | (20) |  |
| Socio-Emotional | 382 | .76 | 40% | 353 | .66 | 67% | <0.001 |
|  |  |  | (24) |  |  | (24) |  |
| Emergent Literacy | 382 | .73 | 35% | 353 | .73 | 73% | <0.001 |
|  |  |  | (18) |  |  | (18) |  |
| Emergent Numeracy | 382 | .76 | 39% | 353 | .71 | 79% | <0.001 |
|  |  |  | (19) |  |  | (16) |  |
| Motor | 382 | .76 | 50% | 353 | .46 | 89% | <0.001 |
|  |  |  | (30) |  |  | (14) |  |
| Executive Function | 382 | .49 | 30% | 353 | .37 | 73% | <0.001 |
|  |  |  | (24) |  |  | (21) |  |
| **Caregiver** |  |  |  |  |  |  |  |
|  |  |  |  |  |  |  |  |
| *Age* | 382 |  |  | 319 |  |  |  |
| <18 – 24 | N/A |  | 5% | N/A |  | 1% |  |
|  |  |  |  |  |  |  |  |
| 25-35 | N/A |  | 64% | N/A |  | 59% |  |
|  |  |  |  |  |  |  |  |
| >36 | N/A |  | 31% | N/A |  | 49% |  |
|  |  |  |  |  |  |  |  |
| *Educational Level* | 379 |  |  | 363 |  |  |  |
| None | N/A |  | 13% | N/A |  | 14% |  |
|  |  |  |  |  |  |  |  |
| Primary | N/A |  | 2% | N/A |  | 47% |  |
|  |  |  |  |  |  |  |  |
| Secondary | N/A |  | 80% | N/A |  | 38% |  |
|  |  |  |  |  |  |  |  |
| Higher | N/A |  | 4% | N/A | N/A | 1% |  |
|  |  |  |  |  |  |  |  |
| *Stimulation* |  |  |  |  |  |  |  |
| Parental stimulation | 373 | .67 | 5.24 | 323 | .72 | 5.29 | 0.76 |
|  |  |  | (2.12) |  |  | (2.25) |  |
| **Context** |  |  |  |  |  |  |  |
| Wealth | 382 |  | 0.04 |  |  |  |  |
|  |  |  | (1.12) |  |  |  |  |
| Toys | 378 | .55 | 4.40 | 322 | .47 | 6.18 | <0.001 |
|  |  |  | (1.86) |  |  | (1.67) |  |
| Books | 364 | .50 | 1.45 | 321 | .56 | 2.04 | <0.001 |
|  |  |  | (1.05) |  |  | (1.12) |  |
|  |  |  |  |  |  |  |  |

## Quasi-experimental Longitudinal Study in Tigray Region in Ethiopia

The Longitudinal study in Tigray Region, Ethiopia (Seiden, Yenew, Kefey, Abrha, & Marino, 2018) was an observational (i.e., non-experimental) study of young children in Ethiopia. One of the goals of the study was to evaluate Save the Children’s Sponsorship Program, which included five components: Early Childhood Care and Development, Basic Education, School Health and Nutrition, Adolescent Development, Maternal and New-born Child Health and Nutrition.

To do so, the study drew representative samples of children in five woredas (i.e., districts) of Save the Children’s Sponsorship impact area and a representative sample of three nearby woredas that were not part of the Sponsorship impact area as a comparison group. A multi-stage cluster sampling procedure was used, taking a random sample of 20 schools in the treatment condition (i.e., Sponsorship impact area) and 20 schools in the control conditions and, within each school, a random sample of eligible children for inclusion in the study. All data were collected in homes and children’s villages in 2017 (baseline or T1) and 2019 (follow-up or T2). No information is available yet about associations between the treatment and child or parental outcomes.

Table S4: Descriptive statistics for Ethiopia

| Variable | T1 | | | T2 | | | T2-T1 |
| --- | --- | --- | --- | --- | --- | --- | --- |
|  | N | α | M(SD) or % | N | α | M(SD) or % | p-value |
| **Child** |  |  |  |  |  |  |  |
| Age in months | 693 |  | 54.80 | 579 |  | 78.30 | <0.001 |
|  |  |  | (9.62) |  |  | (9.29) |  |
| Sex (female = 1) | 693 |  | 49% | 579 |  | 50% |  |
|  |  |  | (50) |  |  | (50) |  |
| **Child Development** |  |  |  |  |  |  |  |
| IDELA (overall) | 693 | .84 | 31% | 579 | .87 | 57% | <0.001 |
|  |  |  | (16) |  |  | (21) |  |
| Socio-Emotional | 693 | .69 | 29% | 579 | .76 | 59% | <0.001 |
|  |  |  | (20) |  |  | (26) |  |
| Emergent Literacy | 693 | .69 | 21% | 579 | .80 | 45% | <0.001 |
|  |  |  | (16) |  |  | (25) |  |
| Emergent Numeracy | 693 | .66 | 35% | 579 | .76 | 63% | <0.001 |
|  |  |  | (18) |  |  | (22) |  |
| Motor | 693 | .61 | 38% | 579 | .65 | 62% | <0.001 |
|  |  |  | (22) |  |  | (24) |  |
| Executive Function | 693 | .51 | 28% | 579 | .46 | 59% | <0.001 |
|  |  |  | (23) |  |  | (28) |  |
| **Caregiver** |  |  |  |  |  |  |  |
|  |  |  |  |  |  |  |  |
| Age | 687 |  |  | 576 |  |  |  |
| <18 – 24 | N/A |  | 11% | N/A |  | 6% |  |
|  |  |  |  |  |  |  |  |
| 25-35 | N/A |  | 56% | N/A |  | 52% |  |
|  |  |  |  |  |  |  |  |
| >36 | N/A |  | 32% | N/A |  | 43% |  |
|  |  |  |  |  |  |  |  |
| Educational Level | 659 |  |  | 560 |  |  |  |
| None | N/A |  | 77% | N/A |  | 75% |  |
|  |  |  |  |  |  |  |  |
| Primary | N/A |  | 22% | N/A |  | 23% |  |
|  |  |  |  |  |  |  |  |
| Secondary | N/A |  | 0% | N/A |  | 1% |  |
|  |  |  |  |  |  |  |  |
| Higher | N/A |  | 1% | N/A |  | 1% |  |
|  |  |  |  |  |  |  |  |
| *Stimulation* |  |  |  |  |  |  |  |
| Parental stimulation | 656 | .86 | 5.72 | 507 | .92 | 7.56 | <0.001 |
|  |  |  | (2.98) |  |  | (2.55) |  |
| **Context** |  |  |  |  |  |  |  |
| Wealth | 647 |  | -0.05 | N/A |  |  |  |
|  |  |  | (1.09) |  |  |  |  |
| Toys | 636 | .75 | 3.50 | 491 | .85 | 4.40 | <0.001 |
|  |  |  | (2.26) |  |  | (2.74) |  |
| Books | 622 | .63 | 1.69 | 498 | .80 | 206 | <0.001 |
|  |  |  | (1.32) |  |  | (1.70) |  |
|  |  |  |  |  |  |  |  |

## Rwanda’s Advancing the Right to Read Program

This study was a quasi-experimental evaluation of the Advancing the Right to Read Program in Rwanda (Iwamoto, Abimpaye, & Mukantagwera, 2019), using difference-in-differences to compare children who received and did not receive the program. The study took a random sample of schools from Gasabo and Ngororero, which were districts receiving the intervention, as well as from neighboring Nyabihu and Kikukiro districts, which were not receiving the intervention. A total of 30 random pre-primary classrooms per treatment arm and 10 children per classroom were selected. Data collection was done by 24 data collectors from Save the Children, who received training in the tools. Save the Children supervised procedures. Data were collected in 2018 (baseline or T1) and 2019 (follow-up or T2).

The results of the study indicated significant associations between receiving the program and child development (i.e., IDELA scores), particularly emergent numeracy and literacy. The study also found associations between the program and parental stimulation.

Table S5: Descriptive statistics for Rwanda

|  |  |  | |  | |  | |  | |  | |  | |  |
| --- | --- | --- | --- | --- | --- | --- | --- | --- | --- | --- | --- | --- | --- | --- |
| Variable | | T1 | | | | | T2 | | | | | | T2-T1 | |
|  |  | N | α | | M(SD) or % | | N | | α | | M(SD) or % | | p-value | |
| **Child** | |  |  | |  | |  | |  | |  | |  | |
| Age in months | | 596 |  | | 53.23 | | 502 | |  | | 69.06 | | <0.001 | |
|  | |  |  | | (6.51) | |  | |  | | (7.03) | |  | |
| Sex(female = 1) | | 596 |  | | 49% | | 502 | |  | | 49% | |  | |
|  | |  |  | | (50) | |  | |  | | (50%) | |  | |
| **Child Development** | |  |  | |  | |  | |  | |  | |  | |
| IDELA (overall) | | 596 | .81 | | 31% | | 502 | | .86 | | 62% | | <0.001 | |
|  | |  |  | | (13) | |  | |  | | (17) | |  | |
| Socio-Emotional | | 596 | .68 | | 28% | | 502 | | .69 | | 58% | | <0.001 | |
|  | |  |  | | (18) | |  | |  | | (21) | |  | |
| Emergent Literacy | | 596 | .55 | | 23% | | 502 | | .79 | | 58% | | <0.001 | |
|  | |  |  | | (13) | |  | |  | | (22) | |  | |
| Emergent Numeracy | | 596 | .66 | | 30% | | 502 | | .76 | | 57% | | <0.001 | |
|  | |  |  | | (13) | |  | |  | | (20) | |  | |
| Motor | | 596 | .65 | | 41% | | 502 | | .63 | | 77% | | <0.001 | |
|  | |  |  | | (22) | |  | |  | | (19) | |  | |
| Executive Function | | 596 | .34 | | 43% | | 502 | | .52 | | 60% | | <0.001 | |
|  | |  |  | | (23) | |  | |  | | (25) | |  | |
| **Caregiver** | |  |  | |  | |  | |  | |  | |  | |
|  | |  |  | |  | |  | |  | |  | |  | |
| *Age* | | 552 |  | |  | | 496 | |  | |  | |  | |
| <18 – 24 | | N/A |  | | 5% | | N/A | |  | | 2% | |  | |
|  | |  |  | |  | |  | |  | |  | |  | |
| 25-35 | | N/A |  | | 65% | | N/A | |  | | 63% | |  | |
|  | |  |  | |  | |  | |  | |  | |  | |
| >36 | | N/A |  | | 29% | | N/A | |  | | 34% | |  | |
|  | |  |  | |  | |  | |  | |  | |  | |
| *Educational Level* | | 556 |  | |  | | 499 | |  | |  | |  | |
| None | | N/A |  | | 24% | | N/A | |  | | 38% | |  | |
|  | |  |  | |  | |  | |  | |  | |  | |
| Primary | | N/A |  | | 55% | | N/A | |  | | 43% | |  | |
|  | |  |  | |  | |  | |  | |  | |  | |
| Secondary | | N/A |  | | 14% | | N/A | |  | | 13% | |  | |
|  | |  |  | |  | |  | |  | |  | |  | |
| Higher | | N/A |  | | 7% | | N/A | |  | | 6% | |  | |
|  | |  |  | |  | |  | |  | |  | |  | |
| *Stimulation* | |  |  | |  | |  | |  | |  | |  | |
| Parental stimulation | | 572 | .79 | | 4.04 | | 475 | | .79 | | 4.97 | | <0.001 | |
|  | |  |  | | (2.63) | |  | |  | | (2.72) | |  | |
| **Context** | |  |  | |  | |  | |  | |  | |  | |
| Wealth | | 588 |  | | -0.03 | | N/A | |  | |  | |  | |
|  | |  |  | | (1.03) | |  | |  | |  | |  | |
| Toys | | 579 | .54 | | 2.63 | | 490 | | .64 | | 3.48 | | <0.001 | |
|  | |  |  | | (1.49) | |  | |  | | (1.79) | |  | |
| Books | | 594 | .51 | | 1.12 | | 500 | | .57 | | 1.59 | | <0.001 | |
|  | |  |  | | (0.95) | |  | |  | | (1.14) | |  | |

# Appendix B: Psychometric analysis

## Bangladesh

**
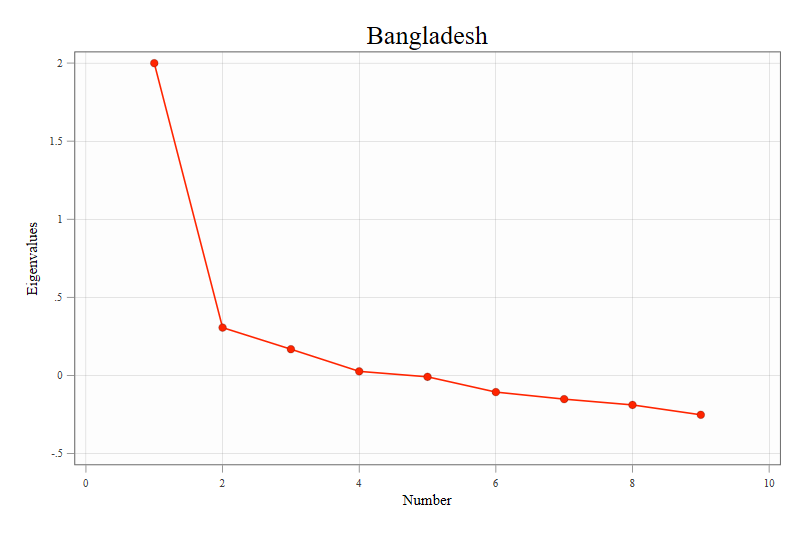
**

Figure S1: Scree plot of eigenvalues for stimulation items - Bangladesh

**
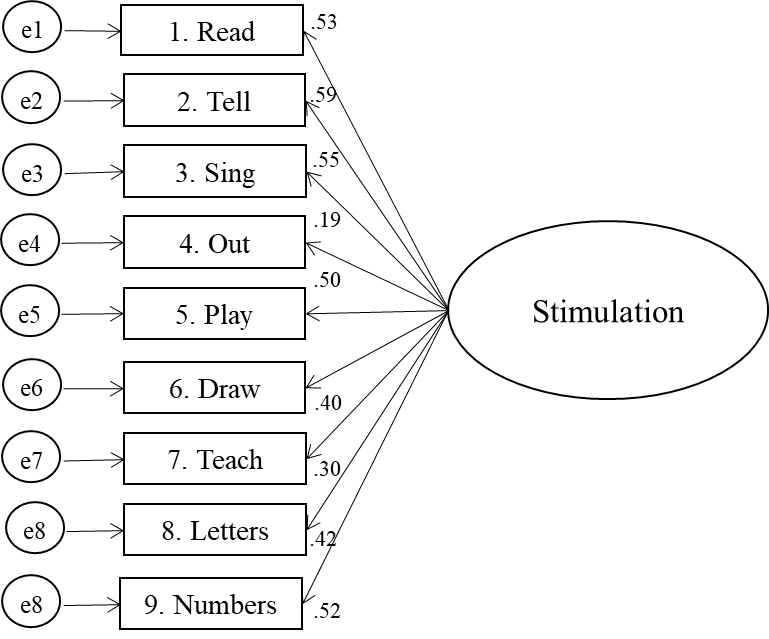
**

Figure S2: Measurement model for caregivers’ stimulation in Bangladesh

Note. The figure presents standardized factor loadings. $RMSEA=0.06;CFI=0.93.;TLI=0.89$. Correlation between latent factor and observed score$=0.97$

**
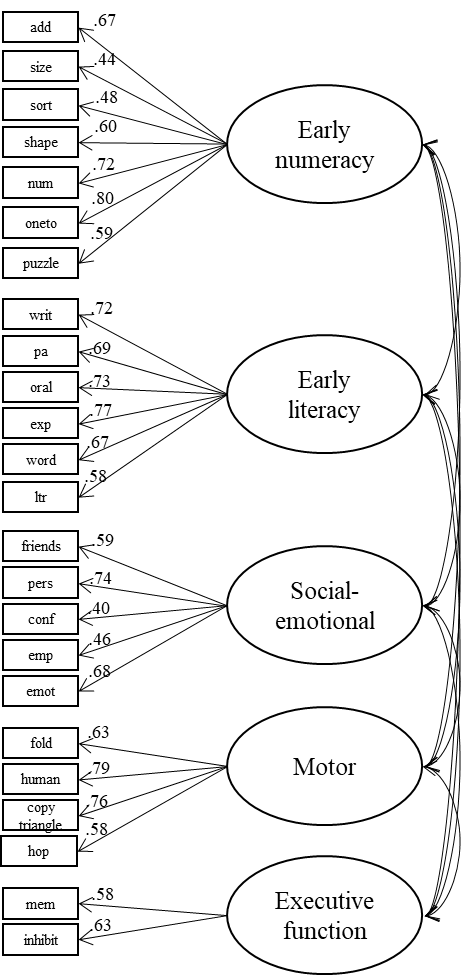
**

Figure S3: Measurement model for IDELA domains in Bangladesh

Note. The figure presents standardized factor loadings. $RMSEA=0.07;CFI=0.91;TLI=0.89$.

## Bhutan

**
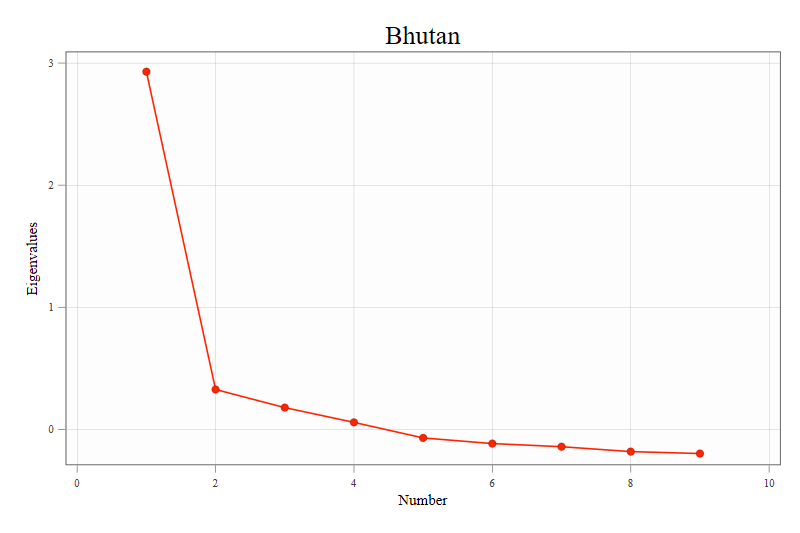
**

Figure S4: Scree plot of eigenvalues for stimulation items - Bhutan

**
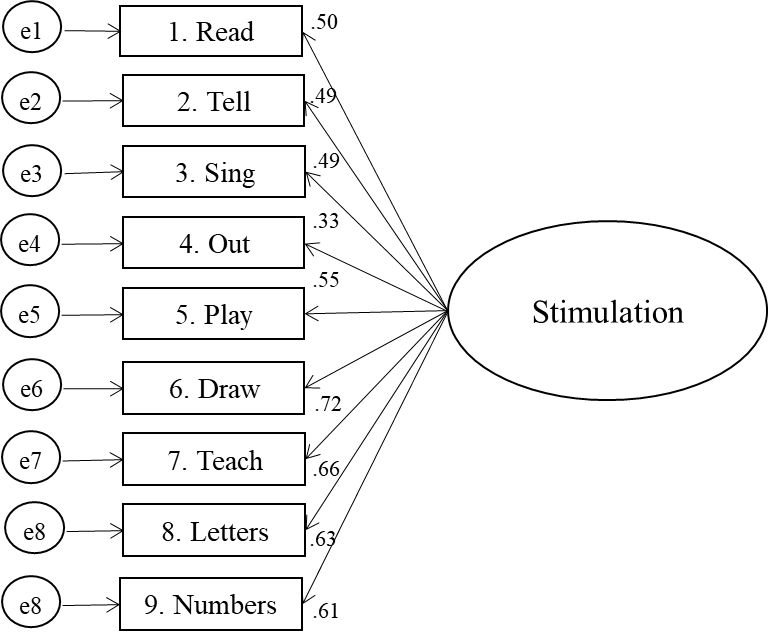
**

Figure S5: Measurement model for caregivers’ stimulation in Bhutan

Note. The figure presents standardized factor loadings. $RMSEA=0.07;CFI=0.94;TLI=0.91$. Correlation between latent factor and observed score$=0.99$

**
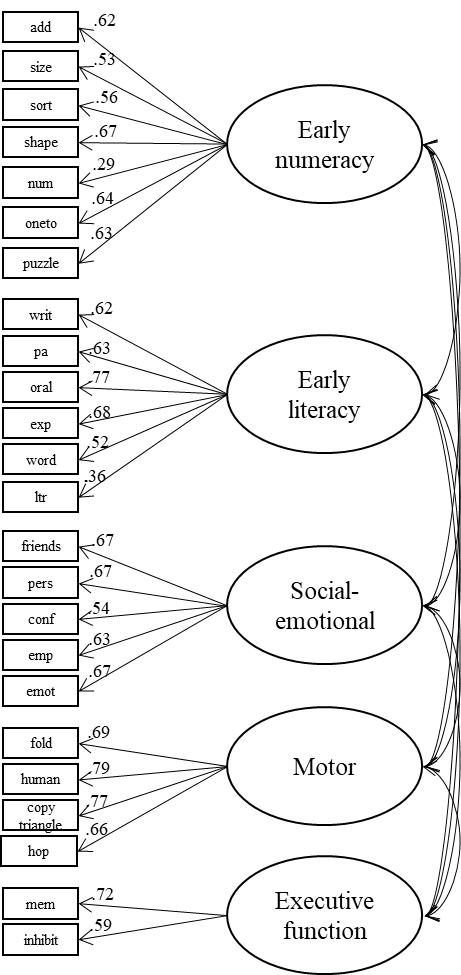
**

Figure S6: Measurement model for IDELA domains in Bhutan

Note. The figure presents standardized factor loadings. $RMSEA=0.06;CFI=0.91;TLI=0.90$.

## Cambodia

**
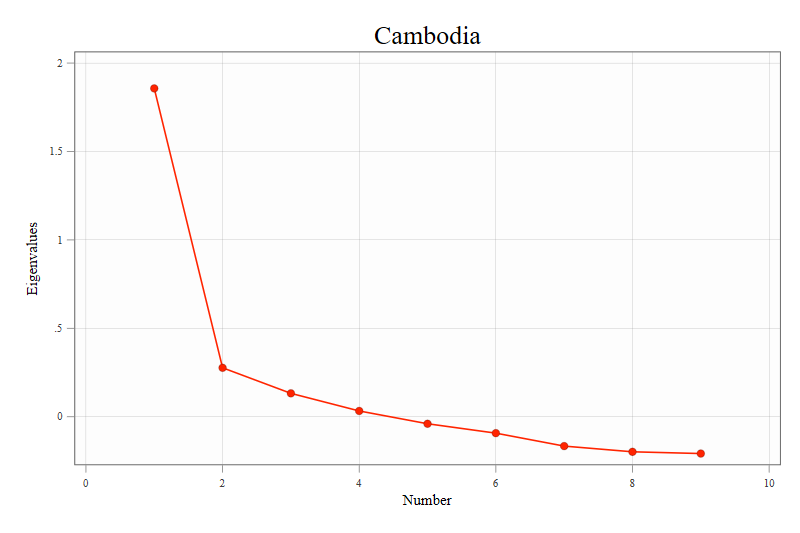
**

Figure S7: Scree plot of eigenvalues for stimulation items - Cambodia

**
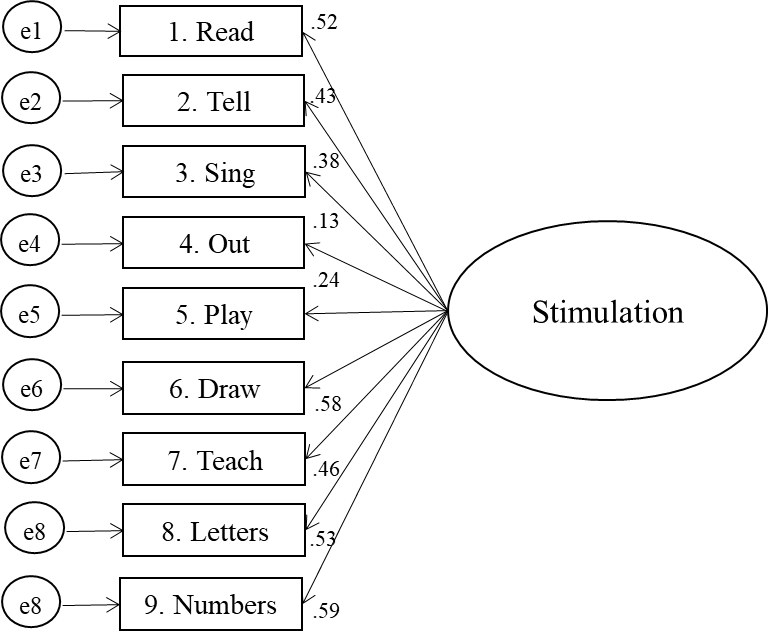
**

Figure S8: Measurement model for caregivers’ stimulation in Cambodia

Note. The figure presents standardized factor loadings. $RMSEA=0.06;CFI=0.92;TLI=0.89$. Correlation between latent factor and observed score$=0.97$

**
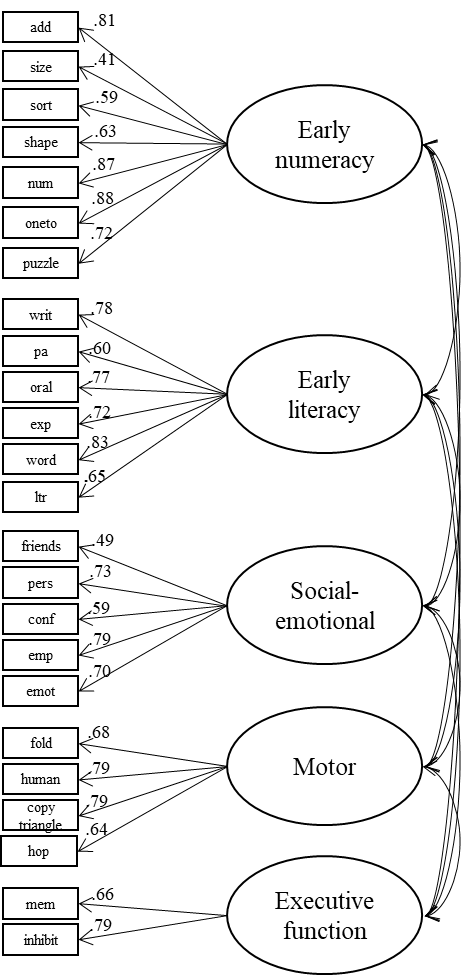
**

Figure S9: Measurement model for IDELA domains in Cambodia

Note. The figure presents standardized factor loadings. $RMSEA=0.07;CFI=0.90;TLI=0.89$.

## Ethiopia

**
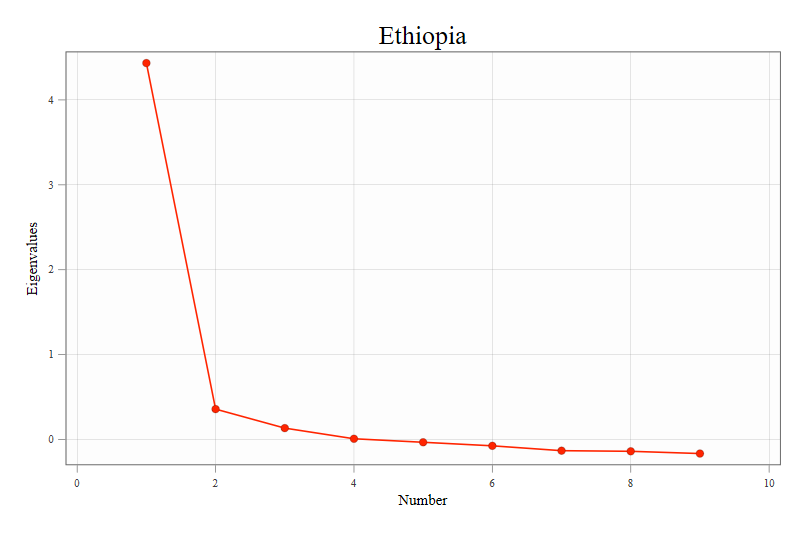
**

Figure S10: Scree plot of eigenvalues for stimulation items - Ethiopia

**
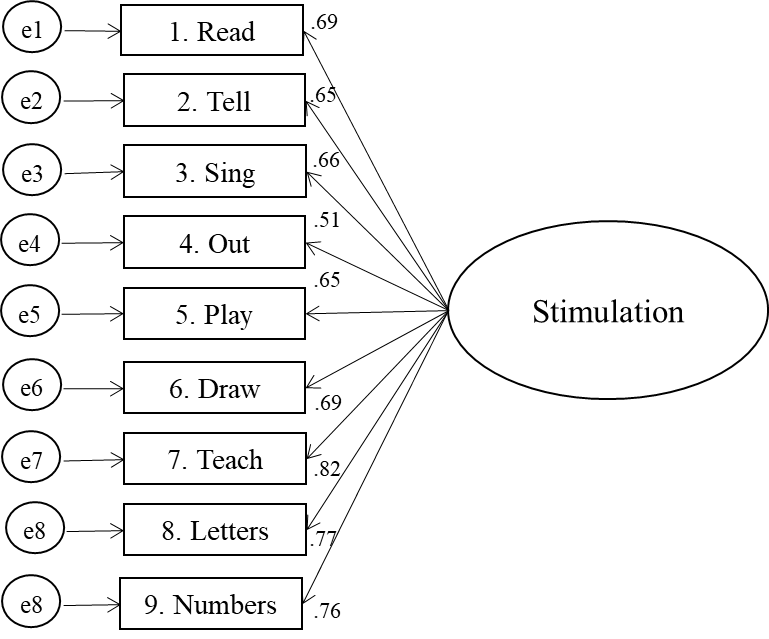
**

Figure S11: Measurement model for caregivers’ stimulation in Ethiopia

Note. The figure presents standardized factor loadings. $RMSEA=0.08;CFI=0.96;TLI=0.94$. Correlation between latent factor and observed score$=0.99$

**
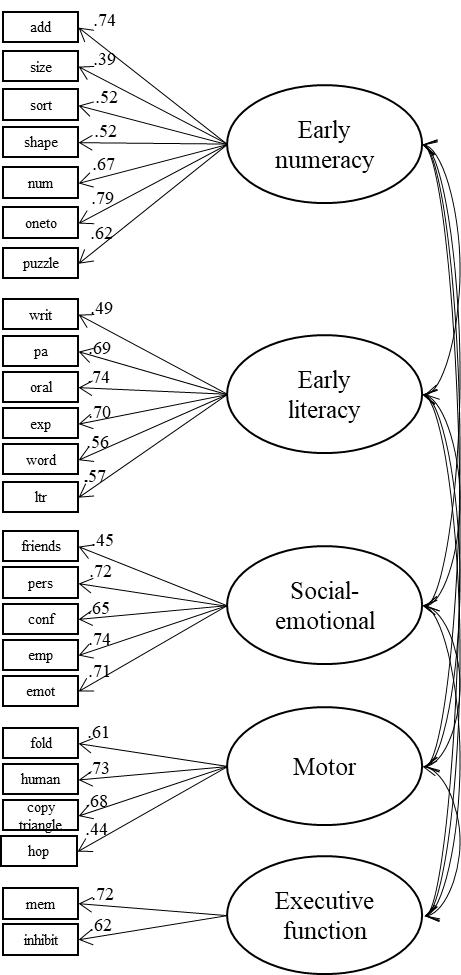
**

Figure S12: Measurement model for IDELA domains in Ethiopia

Note. The figure presents standardized factor loadings. $RMSEA=0.07;CFI=0.89;TLI=0.88$.

## Rwanda

**
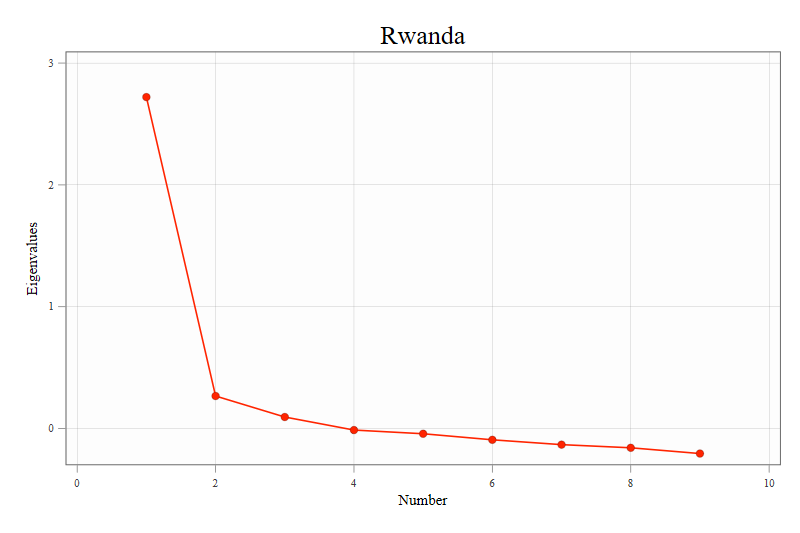
**

Figure S13: Scree plot of eigenvalues for stimulation items - Rwanda

**
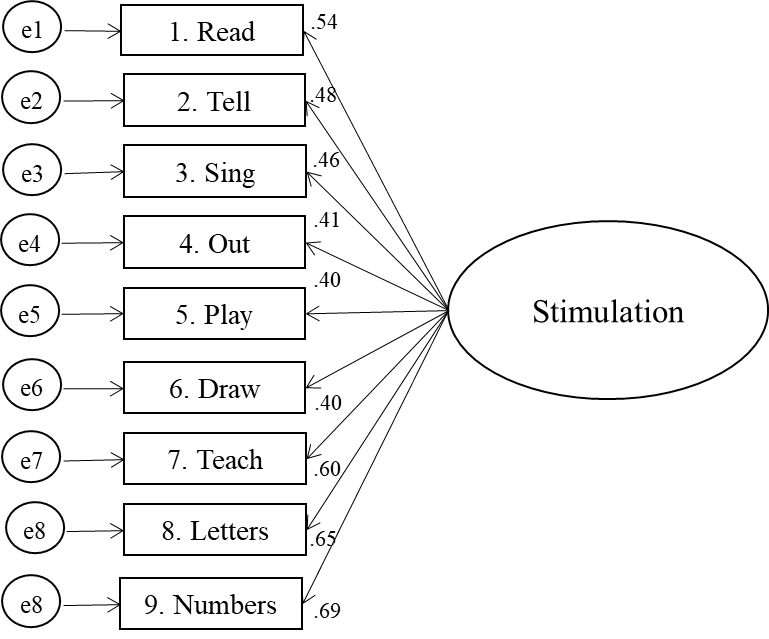
**

Figure S14: Measurement model for caregivers’ stimulation in Rwanda

Note. The figure presents standardized factor loadings. $RMSEA=0.07;CFI=0.93;TLI=0.90$. Correlation between latent factor and observed score$=0.98$


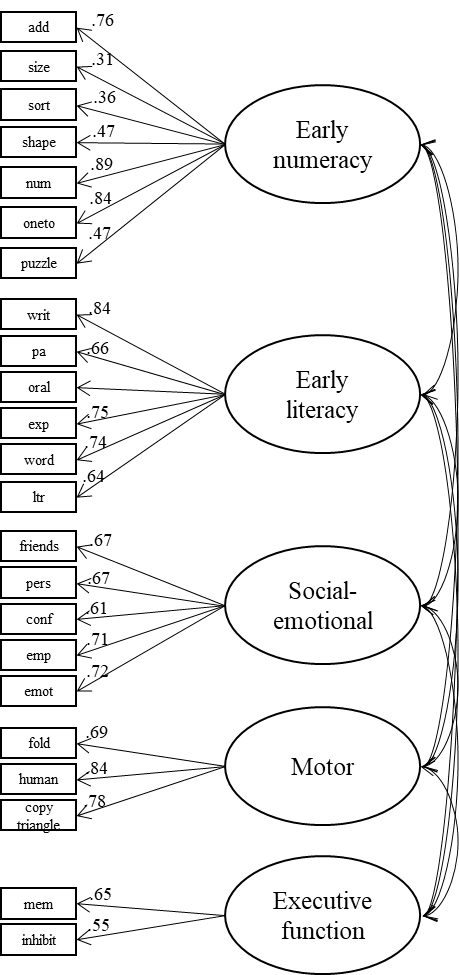


Figure S15: Measurement model for IDELA domains in Rwanda

Note. The figure presents standardized factor loadings. $RMSEA=0.07;CFI=0.92;TLI=0.91$.

# Appendix C. wealth index

Table S6. Indicators used to construct country-specific wealth indices.

| Items | Bangladesh | Bhutan | Cambodia | Ethiopia | Rwanda |
| --- | --- | --- | --- | --- | --- |
| Roof material | X | X |  |  |  |
| Wall material | X | X |  |  |  |
| Toilet inside dwelling | X | X |  | X |  |
| Kitchen |  | X |  | X |  |
| Rooms (#) |  | X |  | X |  |
| Electricity | X | X | X | X | X |
| Water |  | X |  |  |  |
| Radio |  | X | X | X | X |
| TV |  | X | X | X | X |
| Fridge |  | X |  |  |  |
| Computer |  | X |  |  |  |
| Bicycle |  |  |  |  | X |
| Car |  | X |  |  |  |
| Washing machine | | X | X |  |  |
| Microwave |  | X |  |  |  |
| Land |  | X |  | X | X |
| Livestock |  | X |  | X | X |
| Cellphone |  |  | X | X | X |
| Wardrobe |  |  | X |  |  |
| DVD |  |  | X |  |  |
| Battery |  |  | X |  |  |

# Appendix D. Full results

Table S7. RE models for the association between stimulation and ECD using pooled data

| VARIABLES | | Numeracy | Literacy | Social-emotional | Motor | EF |
| --- | --- | --- | --- | --- | --- | --- |
|  |  |  |  |  |  |  |
| Stimulation | | 0.70*** | 0.75*** | 0.76*** | 0.71*** | 0.58*** |
|  | | (0.10) | (0.11) | (0.11) | (0.13) | (0.13) |
| Child is female | | 0.01 | 2.40*** | 0.93* | 2.67*** | 1.12* |
|  | | (0.42) | (0.46) | (0.47) | (0.55) | (0.56) |
| Child age in months | | 0.67*** | 0.73*** | 0.51*** | 0.97*** | 0.82*** |
|  | | (0.03) | (0.03) | (0.03) | (0.04) | (0.04) |
| Mother’s age | |  |  |  |  |  |
|  | <18 – 24 years ^a^ | - | - | - | - | - |
|  |  |  |  |  |  |  |
|  | 25 – 35 years | -0.24 | -0.96 | 1.13+ | 0.31 | 0.17 |
|  |  | (0.58) | (0.62) | (0.64) | (0.77) | (0.77) |
|  | >36 years | 0.37 | -1.14 | 0.59 | -0.09 | 0.69 |
|  |  | (0.75) | (0.80) | (0.83) | (0.98) | (1.01) |
| Mother’s education | |  |  |  |  |  |
|  | None ^a^ | - | - | - | - | - |
|  |  |  |  |  |  |  |
|  | Primary | 0.04 | 0.06 | 0.15 | -0.05 | -0.48 |
|  |  | (0.66) | (0.69) | (0.71) | (0.84) | (0.90) |
|  | Secondary | 1.87** | 1.12+ | 0.83 | 0.88 | 1.15 |
|  |  | (0.65) | (0.68) | (0.71) | (0.86) | (0.91) |
|  | Higher | 1.97* | 1.87+ | -0.74 | -0.79 | 2.46+ |
|  |  | (0.89) | (0.97) | (0.95) | (1.19) | (1.26) |
|  | Non-formal | 1.81 | -0.27 | -2.83+ | -3.16 | 4.40* |
|  |  | (1.39) | (1.45) | (1.55) | (2.06) | (2.22) |
| Wealth | | 0.32 | 0.92*** | -0.04 | 0.99*** | 0.49+ |
|  | | (0.21) | (0.23) | (0.24) | (0.28) | (0.29) |
| Books | | 1.84*** | 2.32*** | 1.52*** | 2.39*** | 1.97*** |
|  | | (0.21) | (0.21) | (0.23) | (0.27) | (0.27) |
| Toys | | 2.48*** | 2.99*** | 2.73*** | 3.23*** | 2.63*** |
|  | | (0.15) | (0.16) | (0.16) | (0.18) | (0.19) |
| Treatment (=1) | | 2.38*** | 2.72*** | 2.77*** | 2.96*** | 1.89** |
|  | | (0.45) | (0.48) | (0.50) | (0.59) | (0.59) |
| Country FE | |  |  |  |  |  |
|  | Bangladesh ^a^ | - | - | - | - | - |
|  |  |  |  |  |  |  |
|  | Bhutan | -9.41*** | -15.62*** | -7.87*** | -15.40*** | -2.67** |
|  |  | (0.61) | (0.66) | (0.66) | (0.81) | (0.83) |
|  | Cambodia | 9.97*** | 7.73*** | 11.27*** | 5.35*** | 0.06 |
|  |  | (0.87) | (0.92) | (1.08) | (1.09) | (1.13) |
|  | Ethiopia | 5.83*** | -6.06*** | 5.95*** | -5.15*** | -1.34 |
|  |  | (0.89) | (0.93) | (0.96) | (1.10) | (1.12) |
|  | Rwanda | 6.38*** | 7.13*** | 10.85*** | 10.16*** | 12.89*** |
|  |  | (0.80) | (0.86) | (0.89) | (0.97) | (1.05) |
|  |  |  |  |  |  |  |
|  | Observations | 9,808 | 9,808 | 9,808 | 9,808 | 9,808 |
|  | Number of children | 4,904 | 4,904 | 4,904 | 4,904 | 4,904 |

*Note*. Robust standard errors in parentheses.

^a^ Omitted category

+ *p*<.1, * *p*<.05, ** *p*<.01, *** *p*<.001

Table S8. Summary of FE models for the association between stimulation and ECD using pooled data

| VARIABLES | Numeracy | Literacy | Social-emotional | Motor | EF |
| --- | --- | --- | --- | --- | --- |
|  |  |  |  |  |  |
| Stimulation | 0.80*** | 0.87*** | 1.07*** | 0.83*** | 0.79*** |
|  | (0.16) | (0.17) | (0.17) | (0.20) | (0.19) |
| Books | 3.18*** | 3.74*** | 2.67*** | 3.81*** | 2.59*** |
|  | (0.31) | (0.33) | (0.35) | (0.41) | (0.40) |
| Toys | 3.82*** | 4.49*** | 4.25*** | 4.90*** | 3.94*** |
|  | (0.21) | (0.22) | (0.23) | (0.26) | (0.26) |
|  |  |  |  |  |  |
| Observations | 9,808 | 9,808 | 9,808 | 9,808 | 9,808 |
| Number of children | 4,904 | 4,904 | 4,904 | 4,904 | 4,904 |

*Note*. Robust standard errors in parentheses.

+ *p*<.1, * *p*<.05, ** *p*<.01, *** *p*<.001

# Appendix E. Results for models with standardized variables

Table S9. Summary of RE models for the association between stimulation and ECD using pooled data (standardized)

| VARIABLES | | Numeracy | Literacy | Social-emotional | Motor | EF |
| --- | --- | --- | --- | --- | --- | --- |
|  |  |  |  |  |  |  |
| Stimulation | | 0.08*** | 0.08*** | 0.08*** | 0.06*** | 0.05*** |
|  |  | (0.01) | (0.01) | (0.01) | (0.01) | (0.01) |
| Child is female | | 0.00 | 0.09*** | 0.04* | 0.09*** | 0.04* |
|  |  | (0.02) | (0.02) | (0.02) | (0.02) | (0.02) |
| Child age in months | | 0.03*** | 0.03*** | 0.02*** | 0.03*** | 0.03*** |
|  |  | (0.00) | (0.00) | (0.00) | (0.00) | (0.00) |
| Mother’s age | |  |  |  |  |  |
|  | <18 – 24 years ^a^ | - | - | - | - | - |
|  |  |  |  |  |  |  |
|  | 25 – 35 years | -0.01 | -0.04 | 0.05+ | 0.01 | 0.01 |
|  |  | (0.03) | (0.02) | (0.03) | (0.03) | (0.03) |
|  | >36 years | 0.02 | -0.04 | 0.02 | -0.00 | 0.02 |
|  |  | (0.03) | (0.03) | (0.03) | (0.03) | (0.04) |
| Mother’s education | |  |  |  |  |  |
|  | None ^a^ | - | - | - | - | - |
|  |  |  |  |  |  |  |
|  | Primary | 0.00 | 0.00 | 0.01 | -0.00 | -0.02 |
|  |  | (0.03) | (0.03) | (0.03) | (0.03) | (0.03) |
|  | Secondary | 0.08** | 0.04 | 0.03 | 0.03 | 0.04 |
|  |  | (0.03) | (0.03) | (0.03) | (0.03) | (0.03) |
|  | Higher | 0.08* | 0.07+ | -0.03 | -0.03 | 0.09+ |
|  |  | (0.04) | (0.04) | (0.04) | (0.04) | (0.04) |
|  | Non-formal | 0.08 | -0.01 | -0.11+ | -0.10 | 0.16* |
|  |  | (0.06) | (0.06) | (0.06) | (0.07) | (0.08) |
| Wealth | | 0.01 | 0.04*** | -0.00 | 0.03*** | 0.02+ |
|  |  | (0.01) | (0.01) | (0.01) | (0.01) | (0.01) |
| Books | | 0.11*** | 0.13*** | 0.09*** | 0.11*** | 0.10*** |
|  |  | (0.01) | (0.01) | (0.01) | (0.01) | (0.01) |
| Toys | | 0.23*** | 0.24*** | 0.23*** | 0.22*** | 0.20*** |
|  |  | (0.01) | (0.01) | (0.01) | (0.01) | (0.01) |
| Treatment (=1) | | 0.10*** | 0.11*** | 0.11*** | 0.10*** | 0.07** |
|  | | (0.02) | (0.02) | (0.02) | (0.02) | (0.02) |
| Country FE | |  |  |  |  |  |
|  | Bangladesh ^a^ | - | - | - | - | - |
|  |  |  |  |  |  |  |
|  | Bhutan | -0.41*** | -0.61*** | -0.32*** | -0.50*** | -0.10** |
|  |  | (0.03) | (0.03) | (0.03) | (0.03) | (0.03) |
|  | Cambodia | 0.43*** | 0.30*** | 0.45*** | 0.17*** | 0.00 |
|  |  | (0.04) | (0.04) | (0.04) | (0.04) | (0.04) |
|  | Ethiopia | 0.25*** | -0.24*** | 0.23*** | -0.17*** | -0.05 |
|  |  | (0.04) | (0.04) | (0.04) | (0.04) | (0.04) |
|  | Rwanda | 0.27*** | 0.27*** | 0.43*** | 0.33*** | 0.45*** |
|  |  | (0.03) | (0.03) | (0.04) | (0.03) | (0.04) |
|  |  |  |  |  |  |  |
|  | Observations | 9,808 | 9,808 | 9,808 | 9,808 | 9,808 |
|  | Number of children | 4,904 | 4,904 | 4,904 | 4,904 | 4,904 |

*Note*. Robust standard errors in parentheses. All continuous variables, including the outcomes, are standardized to have a mean of zero and standard deviation of one

^a^ Omitted category

+ *p*<.1, * *p*<.05, ** *p*<.01, *** *p*<.001

Table S10. Summary of FE models for the association between stimulation and ECD using pooled data (standardized)

| VARIABLES | Numeracy | Literacy | Social-emotional | Motor | EF |
| --- | --- | --- | --- | --- | --- |
|  |  |  |  |  |  |
| Stimulation | 0.09*** | 0.09*** | 0.11*** | 0.07*** | 0.07*** |
|  | (0.02) | (0.02) | (0.02) | (0.02) | (0.02) |
| Books | 0.19*** | 0.20*** | 0.15*** | 0.17*** | 0.13*** |
|  | (0.02) | (0.02) | (0.02) | (0.02) | (0.02) |
| Toys | 0.35*** | 0.37*** | 0.36*** | 0.33*** | 0.29*** |
|  | (0.02) | (0.02) | (0.02) | (0.02) | (0.02) |
|  |  |  |  |  |  |
| Observations | 9,808 | 9,808 | 9,808 | 9,808 | 9,808 |
| Number of children | 4,904 | 4,904 | 4,904 | 4,904 | 4,904 |

*Note*. Robust standard errors in parentheses. All continuous variables, including the outcomes, are standardized to have a mean of zero and standard deviation of one

+ *p*<.1, * *p*<.05, ** *p*<.01, *** *p*<.001

# Appendix F. Results for country-specific models

Table S11. Summary of FE models for the association between stimulation and ECD using pooled data (standardized) in Bhutan

| VARIABLES | Numeracy | Literacy | Social-emotional | Motor | EF |
| --- | --- | --- | --- | --- | --- |
|  |  |  |  |  |  |
| Stimulation | 0.07* | 0.10** | 0.14*** | 0.12*** | 0.10** |
|  | (0.03) | (0.03) | (0.03) | (0.03) | (0.03) |
| Books | 0.11** | 0.11** | 0.07+ | 0.12** | 0.04 |
|  | (0.04) | (0.04) | (0.04) | (0.04) | (0.04) |
| Toys | 0.31*** | 0.34*** | 0.33*** | 0.35*** | 0.22*** |
|  | (0.03) | (0.03) | (0.03) | (0.03) | (0.03) |
| Constant | 0.02*** | 0.02*** | 0.02*** | 0.02*** | 0.02*** |
|  | (0.00) | (0.01) | (0.01) | (0.00) | (0.00) |
|  |  |  |  |  |  |
| Observations | 2,754 | 2,754 | 2,754 | 2,754 | 2,754 |
| Number of children | 1,377 | 1,377 | 1,377 | 1,377 | 1,377 |

*Note*. Robust standard errors in parentheses

+ *p*<.1, * *p*<.05, ** *p*<.01, *** *p*<.001

Table S12. Summary of FE models for the association between stimulation and ECD using pooled data (standardized) in Bangladesh

| VARIABLES | Numeracy | Literacy | Social-emotional | Motor | EF |
| --- | --- | --- | --- | --- | --- |
|  |  |  |  |  |  |
| Stimulation | -0.03 | -0.01 | -0.03 | -0.01 | -0.02 |
|  | (0.02) | (0.02) | (0.03) | (0.03) | (0.02) |
| Books | 0.29*** | 0.28*** | 0.25*** | 0.26*** | 0.22*** |
|  | (0.02) | (0.02) | (0.03) | (0.03) | (0.02) |
| Toys | 0.29*** | 0.36*** | 0.35*** | 0.30*** | 0.23*** |
|  | (0.02) | (0.02) | (0.03) | (0.03) | (0.02) |
| Constant | 0.00*** | 0.00** | 0.00** | -0.00+ | 0.00** |
|  | (0.00) | (0.00) | (0.00) | (0.00) | (0.00) |
|  |  |  |  |  |  |
| Observations | 3,712 | 3,712 | 3,712 | 3,712 | 3,712 |
| Number of children | 1,856 | 1,856 | 1,856 | 1,856 | 1,856 |

*Note*. Robust standard errors in parentheses

+ *p*<.1, * *p*<.05, ** *p*<.01, *** *p*<.001

Table S13. Summary of FE models for the association between stimulation and ECD using pooled data (standardized) in Cambodia

| VARIABLES | Numeracy | Literacy | Social-emotional | Motor | EF |
| --- | --- | --- | --- | --- | --- |
|  |  |  |  |  |  |
| Stimulation | -0.06 | -0.04 | -0.07 | 0.01 | -0.11+ |
|  | (0.06) | (0.06) | (0.06) | (0.06) | (0.06) |
| Books | 0.24*** | 0.27*** | 0.14* | 0.20** | 0.20** |
|  | (0.07) | (0.06) | (0.06) | (0.07) | (0.07) |
| Toys | 0.54*** | 0.50*** | 0.42*** | 0.46*** | 0.51*** |
|  | (0.05) | (0.05) | (0.05) | (0.05) | (0.05) |
| Constant | 0.01* | 0.01* | 0.00 | 0.01*** | 0.01* |
|  | (0.00) | (0.00) | (0.00) | (0.00) | (0.00) |
|  |  |  |  |  |  |
| Observations | 764 | 764 | 764 | 764 | 764 |
| Number of children | 382 | 382 | 382 | 382 | 382 |

*Note*. Robust standard errors in parentheses

+ *p*<.1, * *p*<.05, ** *p*<.01, *** *p*<.001

Table S14. Summary of FE models for the association between stimulation and ECD using pooled data (standardized) in Ethiopia

| VARIABLES | Numeracy | Literacy | Social-emotional | Motor | EF |
| --- | --- | --- | --- | --- | --- |
|  |  |  |  |  |  |
| Stimulation | 0.37*** | 0.30*** | 0.39*** | 0.25*** | 0.35*** |
|  | (0.04) | (0.04) | (0.04) | (0.04) | (0.04) |
| Books | 0.06 | 0.05 | 0.03 | 0.02 | 0.05 |
|  | (0.05) | (0.05) | (0.05) | (0.05) | (0.05) |
| Toys | 0.16** | 0.17** | 0.16** | 0.13* | 0.13* |
|  | (0.05) | (0.05) | (0.05) | (0.05) | (0.05) |
| Constant | 0.01*** | 0.01*** | 0.01** | 0.01*** | 0.01*** |
|  | (0.00) | (0.00) | (0.00) | (0.00) | (0.00) |
|  |  |  |  |  |  |
| Observations | 1,386 | 1,386 | 1,386 | 1,386 | 1,386 |
| Number of children | 693 | 693 | 693 | 693 | 693 |

*Note*. Robust standard errors in parentheses

+ *p*<.1, * *p*<.05, ** *p*<.01, *** *p*<.001

Table S15. Summary of FE models for the association between stimulation and ECD using pooled data (standardized) in Rwanda

| VARIABLES | Numeracy | Literacy | Social-emotional | Motor | EF |
| --- | --- | --- | --- | --- | --- |
|  |  |  |  |  |  |
| Stimulation | 0.19*** | 0.18*** | 0.16*** | 0.13** | -0.00 |
|  | (0.05) | (0.05) | (0.05) | (0.05) | (0.04) |
| Books | 0.18*** | 0.20*** | 0.12* | 0.16** | 0.08 |
|  | (0.05) | (0.05) | (0.05) | (0.05) | (0.05) |
| Toys | 0.29*** | 0.33*** | 0.28*** | 0.30*** | 0.24*** |
|  | (0.05) | (0.05) | (0.05) | (0.05) | (0.05) |
| Constant | 0.02*** | 0.02*** | 0.02*** | 0.02*** | 0.01*** |
|  | (0.00) | (0.00) | (0.00) | (0.00) | (0.00) |
|  |  |  |  |  |  |
| Observations | 1,192 | 1,192 | 1,192 | 1,192 | 1,192 |
| Number of children | 596 | 596 | 596 | 596 | 596 |

*Note*. Robust standard errors in parentheses

+ *p*<.1, * *p*<.05, ** *p*<.01, *** *p*<.001

# References

Iwamoto, S., Abimpaye, M., & Mukantagwera, L. (2019). Advancing the school readiness 4-6 program in Rwanda endline report.

Pisani, L., Dib, G., & Khoy, R. (2016). *Cambodia first read endline report*. Retrieved from

Pisani, L., Dyenka, K., Sharma, P., Chhetri, N., Dang, S., Gayleg, K., & Wangdi, C. (2017). Bhutan’s national ECCD impact evaluation: local, national, and global perspectives. *Early Child Development and Care, 187*(10), 1511-1527. doi:10.1080/03004430.2017.1302944

Seiden, J., Yenew, A., Kefey, K., Abrha, H., & Marino, J. (2018). Central Tigray sponsorship longitudinal study of learning and development: baseline results. In: Save the Children.

Spier, E., Vasudevan, S., Kamto, K., Rahman, A., Hossain, N., Nahar, Z., & Khondker, H. (2018). *Bangladesh early years preschool program evaluation*. Retrieved from
